# Molecular requirements for the inter-subunit interaction and kinetochore recruitment of SKAP and Astrin

**DOI:** 10.1038/ncomms11407

**Published:** 2016-04-20

**Authors:** Alexandra Friese, Alex C. Faesen, Pim J. Huis in ‘t Veld, Josef Fischböck, Daniel Prumbaum, Arsen Petrovic, Stefan Raunser, Franz Herzog, Andrea Musacchio

**Affiliations:** 1 Department of Mechanistic Cell Biology, Max Planck Institute of Molecular Physiology, Otto-Hahn-Straße 11, 44227 Dortmund, Germany; 2 Gene Center Munich, Ludwig-Maximilians-Universität München, Feodor-Lynen-Straße 25, 81377 Munich, Germany; 3 Department of Structural Biochemistry, Max Planck Institute of Molecular Physiology, Otto-Hahn-Straße 11, 44227 Dortmund, Germany; 4 Centre for Medical Biotechnology, Faculty of Biology, University Duisburg-Essen, Universitätsstrasse, 45141 Essen, Germany

## Abstract

Accurate chromosome segregation during cell division is crucial for propagating life and protects from cellular transformation. The SKAP:Astrin heterodimer localizes to spindle microtubules and to mature microtubule–kinetochore attachments during mitosis. Depletion of either subunit disrupts spindle structure and destabilizes kinetochore–microtubule attachments. Here, we identify molecular requirements for the inter-subunit interaction of SKAP and Astrin, and discuss requirements for their kinetochore recruitment. We also identify and characterize a microtubule-binding domain in SKAP, distinct from the SXIP motif that mediates end binding (EB) protein binding and plus end tracking, and show that it stimulates the growth-rate of microtubules, possibly through a direct interaction with tubulin. Mutations targeting this microtubule-binding domain impair microtubule plus-end tracking but not kinetochore targeting, and recapitulate many effects observed during depletion of SKAP. Collectively, our studies represent the first thorough mechanistic analysis of SKAP and Astrin, and significantly advance our functional understanding of these important mitotic proteins.

## Abstract (web summary)

SKAP and Astrin form a heterodimer that localizes to spindle microtubules and to mature microtubule-kinetochore attachments during mitosis. Here, the authors identify molecular requirements for the inter-subunit interaction of SKAP and Astrin and kinetochore recruitment.

## Graphical abstract

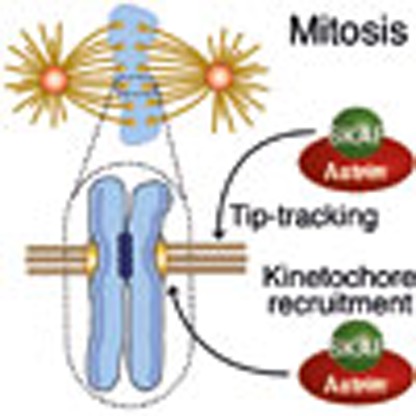

Chromosome segregation requires the coordinated interaction of a sophisticated segregation apparatus on each sister chromatid with a wealth of proteins in the mitotic spindle, including microtubules, microtubule-associated proteins (MAPs) and molecular motors^1^. The segregation apparatus, named the kinetochore, is a multi-subunit assembly that docks on centromeric DNA. It is a complex, multi-layered structured, with ‘inner' and ‘outer' kinetochore domains, respectively, proximal to, and distal from, the chromatin^2^. The outer kinetochore domain mediates interactions with spindle microtubules and associated proteins. Initial kinetochore attachments are established with the side of microtubules (lateral attachments) and are sufficient for congression to the equatorial plane of the spindle^3^,^4^. These attachments then mature to become ‘end-on', with microtubules terminating perpendicularly into the outer kinetochore plate. Conversion from lateral to end-on attachment is a regulated process that involves significant changes in the composition and posttranslational modifications of the outer kinetochore^1^,^4^.

Small kinetochore-associated protein (SKAP, also called Kinastrin or TRAF4-associated factor 1) and Astrin (also named SPAG5 or MAP126) were independently identified, respectively, as the product of a gene (*FLJ14502*) undergoing transcriptional induction at the G_2_/M transition of the cell cycle, and as a spindle-associated protein and a component of sperm flagella^5^,^6^,^7^,^8^,^9^,^10^. It was later realized that SKAP and Astrin form a tight complex (the SKAP:Astrin complex), which may also include sub-stoichiometric amounts of the dynein light chain LC8, Polo-like kinase 1 (Plk1) and Shugoshin 2 (Sgo2; refs 11, 12).

During mitosis, SKAP and Astrin display identical localization patterns. They associate with the spindle and the spindle poles in prophase and prometaphase, and become also recruited to the outer kinetochores but only at the late stages of metaphase and telophase^6^,^7^,^11^,^12^,^13^,^14^, when end-on attachments prevail. In earlier phases of mitosis, Aurora B kinase, a crucial regulator of kinetochore–microtubule interactions^15^, counteracts kinetochore recruitment of the SKAP:Astrin complex^11^,^13^. Thus, the presence of SKAP:Astrin at kinetochores signals the establishment of mature, end-on attachments, where SKAP:Astrin is believed to suppress kinetochore–microtubule turnover^13^,^16^. Finally, the SKAP:Astrin complex has been shown to track the growing plus ends of microtubules^12^,^17^,^18^.

To explain the complex localization pattern of the SKAP:Astrin complex and its functional contributions to chromosome alignment, a number of physical interactions have been invoked. Plus-end tracking was shown to require a serine-X-isoleucine-proline (SXIP, where X is any amino acid) motif in the N-terminal region of SKAP (Fig. 1a; refs 17, 18). SXIP motifs mediate physical interaction of many MAPs with end binding plus-end tracking proteins^19^,^20^, and indeed SKAP:Astrin binds end binding 1 and end binding 3 (EB1 and EB3; refs 17, 18). SKAP may stabilize microtubule plus-end dynamics at kinetochores through an interaction with CLASP1, effectively switching off Kif2b, a member of the kinesin-13 family of microtubule depolymerases, whose abundance at kinetochores is anti-correlated with that of SKAP (ref. 13). Two microtubule motors, CENP-E and Kif18a, have been implicated in kinetochore recruitment of SKAP:Astrin^13^,^21^. Furthermore, subunits of the Knl1-complex, Mis12 complex, Ndc80-complex (KMN) protein network in the outer kinetochore^22^ were also shown to be required for kinetochore localization of SKAP:Astrin in human cells, possibly through a direct interaction^11^,^18^. The precise mechanism of SKAP:Astrin localization to spindle poles, on the other hand, is unknown, but the complex has been implicated in the recruitment of Aurora A kinase to spindle poles^23^.

Biochemical reconstitution of biological interactions is instrumental to grasp the molecular basis of macromolecular function. As there has been little progress until now on the *in vitro* biochemical reconstitution of the SKAP:Astrin complex and of its functions, we decided to apply a combination of *in vitro* approaches and biological validation to explore how SKAP:Astrin interact with themselves and with microtubules and kinetochores. Here, we characterize the inter-subunit interaction of SKAP and Astrin, and the requirements for their kinetochore recruitment. We also identify a microtubule-binding domain in SKAP and show that it works together with an SXIP motif to promote microtubule plus-end tracking.

## Results

### Identification of a minimal SKAP:Astrin interaction domain

**Figure 1:**
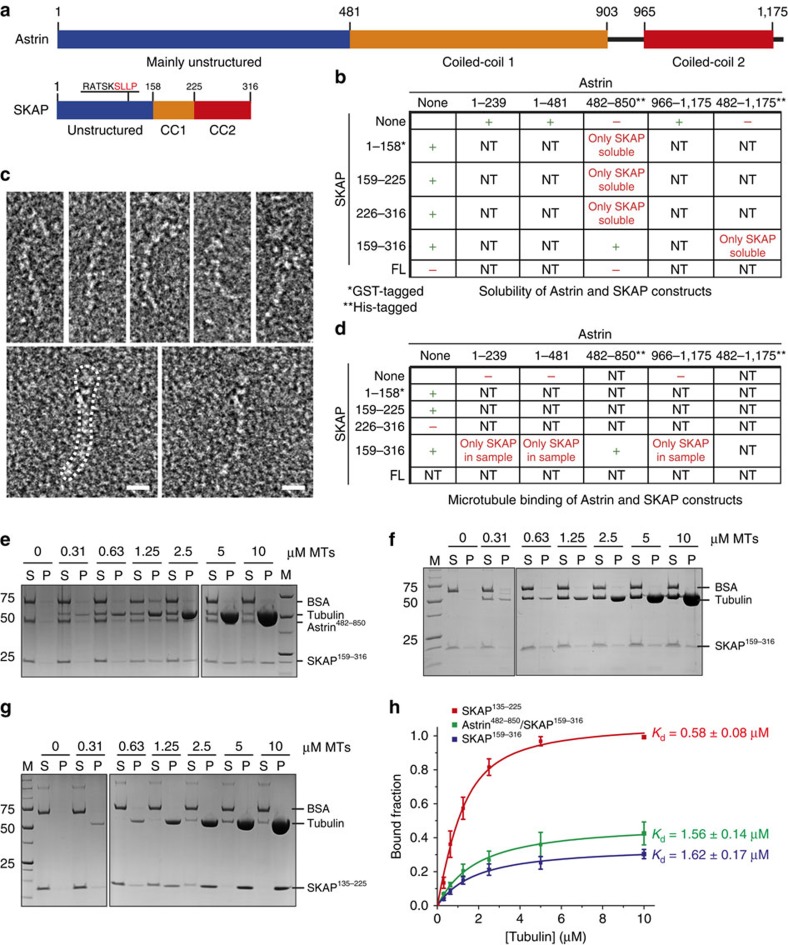
Domain characterization of the SKAP:Astrin complex. (**a**) Domain organization of the SKAP:Astrin complex. Both Astrin and SKAP consist of three domains: a mainly unstructured N-terminal domain followed by two coiled-coil domains. (**b**) Solubility of Astrin and SKAP constructs in isolated and co-expression in bacteria and insect cells. NT, not tested. Samples not marked by asterisks were purified by means of affinity tags that were later removed proteolytically, as discussed in Methods section. (**c**) Representative electron micrographs of negative-stained SKAP^159–316^:Astrin^482–850^ complex. Scale bar, 10 nm. (**d**) Summary of microtubule-binding properties of Astrin and SKAP constructs in co-sedimentation assays with purified isolated or combined constructs. NT, not tested. (**e**) Representative SDS–PAGE of microtubule co-sedimentation assay with 0-10 μM taxol-stabilized microtubules (MTs) and 1 μM SKAP^159–316^:Astrin^482–850^ complex. M, molecular weight marker; P, pellet fraction; S, soluble fraction. Images originate from two different gels as indicated by dividing lines. (**f**) Representative SDS–PAGE of microtubule co-sedimentation assay with 0-10 μM taxol-stabilized MTs and 1 μM SKAP^159–316^. M, molecular weight marker; P, pellet fraction; S, soluble fraction. Images originate from two different gels as indicated by dividing lines. (**g**) Representative SDS–PAGE of microtubule co-sedimentation assay with 0-10 μM taxol-stabilized MTs and 1 μM SKAP^135–225^. M, molecular weight marker; P, pellet fraction; S, soluble fraction. Images originate from two different gels as indicated by dividing lines. (**h**) Quantification and fitting analysis of microtubule co-sedimentation assays shown in (**e**–**g**; mean±s.e.m., *n*≥3).

The structural organization of Astrin and SKAP is shown in Fig. 1a. In line with previous analyses, the COILS server^24^ predicts that both proteins contain extended coiled-coil segments. REPPER, an alternative coiled-coil prediction server that uses a distinct algorithm^25^, did not identify in SKAP or Astrin the regular seven-residue repeat typical of extended coiled coils such as those in the Myosin heavy chain, suggesting that the coiled-coil segments of SKAP and Astrin may contain frequent interruptions.

To identify interaction domains within the human SKAP:Astrin complex, we purified recombinant fragments of SKAP and Astrin that were systematically expressed in isolation of by co-expression in bacteria or insect cells. A C-terminal segment of SKAP (SKAP^159–316^), corresponding to the first and second predicted coiled-coil regions, was found to interact with the first coiled-coil region of Astrin (Astrin^482–850^). This result is consistent with a previous analysis in which segments with similar boundaries were expressed in mammalian cells and subjected to immunoprecipitation analysis^12^. Thus, SKAP^159–316^:Astrin^482–850^ interact directly in the absence of other partners. Co-expression with Dynein Light Chain (LC8) did not rescue the lack of solubility of full-length SKAP or Astrin.

By negative stain electron microscopy (Fig. 1c), the SKAP^159–316^:Astrin^482–850^ complex appeared very elongated, with an overall length of ∼40–50 nm (a larger field of view is shown in Supplementary Fig. 1). This result is expected based on the extensive content of coiled-coil predicted for both interacting segments. Due to its considerable flexibility and lack of structural homogeneity, however, the sample was deemed unsuitable for single-particle analysis or high-resolution cryo-electron microscopy. Previously, a rotary shadowing electron microscopy analysis of full-length Astrin that had been refolded from bacterial inclusion bodies was shown to resemble an 80-nm ‘lollypop', with a 65-nm rod terminating at one end in a ‘head' domain, surmised to correspond to the N-terminal region of Astrin^8^. The apparent twofold symmetry of the particle led to conclude that Astrin might form a dimeric, parallel coiled-coil^8^. Our attempts to verify the oligomerization state of the SKAP^159–316^:Astrin^482–850^ complex in analytical ultracentrifugation (AUC) sedimentation velocity experiments were thwarted by limited sample solubility.

### A minimal microtubule-binding domain in SKAP

It has been proposed that the N-terminal region of SKAP, and the SXIP motif within it, is the major determinant of SKAP localization to the mitotic spindle^17^,^18^. However, a deletion construct encompassing residues 159–317 of SKAP, which lacks the SXIP motif, mediated SKAP localization to the mitotic spindle, albeit to a lower degree in comparison to full-length SKAP, while a construct encompassing residues 1–158 failed to localize to the spindle^12^. These observations suggest that besides binding microtubules via end binding proteins, SKAP may also contain a region for direct targeting to microtubules. Indeed full-length SKAP, but not a C-terminal segment encompassing residues 167–316, associated with microtubules in co-sedimentation assays *in vitro*^11^.

To investigate this problem further, we asked if SKAP^159–316^ or the SKAP^159–316^:Astrin^482–850^ complex interacted with microtubules. In co-sedimentation experiments, the SKAP^159–316^:Astrin^482–850^ complex interacted with taxol-stabilized microtubules with an apparent dissociation constant (*K*_d_) of ∼1.6 μM (Fig. 1e and 1h, summarized in Fig. 1d). SKAP^159–316^ interacted with microtubules with essentially identical affinity in the absence of Astrin (Fig. 1f,1h), indicating that Astrin^482–850^ is not required for the interaction with microtubules and that the observed interaction with microtubules is mediated by SKAP. Furthermore, neither Astrin^1–239^ nor Astrin^966–1,175^ bound to microtubules *in vitro* (Fig. 1d and Supplementary Fig. 2A), suggesting that Astrin does not contribute to microtubule binding.

We refined our analysis of the microtubule-binding domain of SKAP. SKAP^226–316^ did not bind microtubules (Fig. 1d), while SKAP^159–225^ showed a *K*_d_ for microtubules of ∼2.5 μM, only ∼1.5x lower than SKAP^159–316^'s (Supplementary Fig. 2B). Thus, residues 226–316 of SKAP are neither sufficient nor necessary for microtubule binding. Conversely, extension towards the N-terminus (in SKAP^135–225^) showed the highest binding affinity for microtubules (∼0.58 μM; Fig. 1g,1h). Further extension of this segment towards the SKAP N-terminus (SKAP^103–225^) did not lead to further increases in binding affinity (Supplementary Fig. 2B). Thus, the microtubule-binding domain of SKAP extends N-terminally beyond previously tested boundaries (ending at residues 157 or 167)^11^,^12^.

### Dissection of the microtubule-binding mechanism

**Figure 2:**
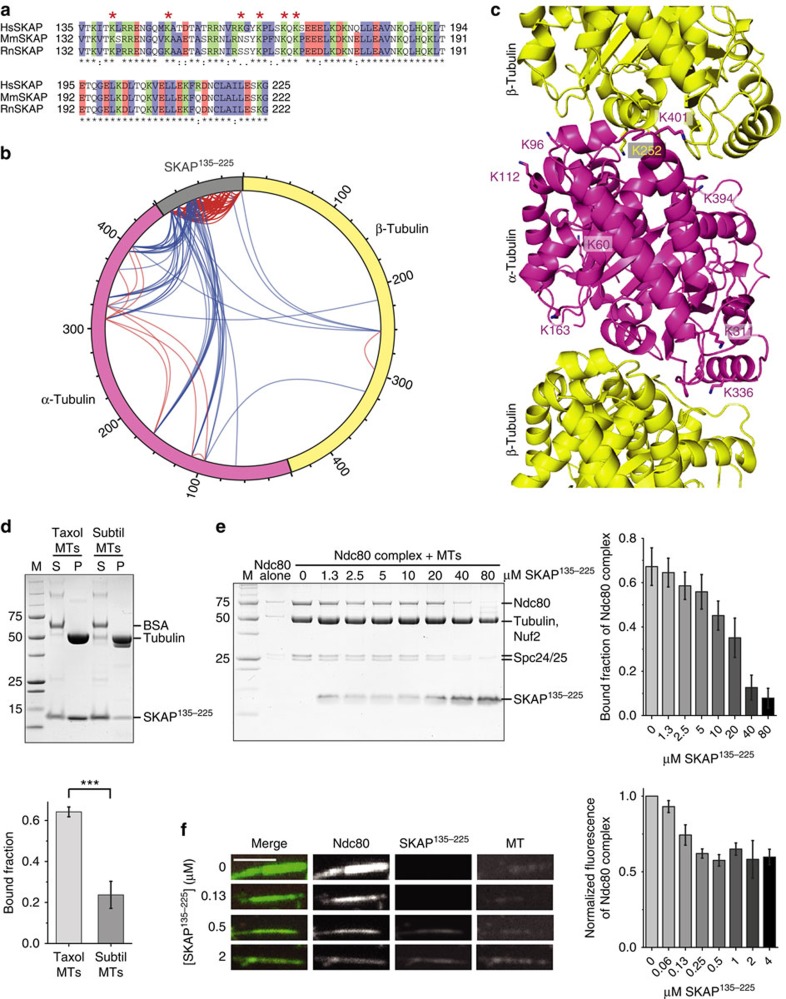
SKAP binds microtubules at the α-/β-tubulin interface and competes with the Ndc80 complex for microtubule binding. (**a**) Sequence alignment of SKAP^135–225^ showing amino acid conservation between *Homo sapiens*, *Mus musculus* and *Rattus norvegicus*. Asterisks indicate amino acids identified in cross-linking analysis with microtubules. (**b**) Intermolecular (blue) and intramolecular (red) cross-links found between SKAP^135–225^, α-1B and β-2B tubulin in cross-linking reactions with 10 μM SKAP^135–225^ and 10 μM taxol-stabilized microtubules. (**c**) Cartoon representation of tubulin (PDB ID: 3RYC) showing residues involved in cross-linking with SKAP^135–225^. Respective residues are highlighted in stick representation. (**d**) Representative SDS–PAGE (top) and quantification (bottom) of microtubule co-sedimentation assays with 3 μM SKAP^135–225^ and 3 μM taxol-stabilized or subtilisin-treated microtubules (mean±s.e.m., *n*=3, *t*-test: ***P<0.005). M, molecular weight marker; P, pellet fraction; S, soluble fraction. (**e**) Representative SDS–PAGE of the pellet fraction (left) and quantification (right) of microtubule co-sedimentation competition assay with 1 μM taxol-stabilized microtubules and 1 μM Ndc80 complex in presence of 0–80 μM SKAP^135–225^ (mean±s.e.m., *n*=3). (**f**) Representative images (left) and quantification (right) of fluorescence microtubule flow cell assay with 100 nM taxol-stabilized, HiLyte-647 labelled microtubules, 35 nM GFP-tagged Ndc80 complex and 0–4 μM mCherry-SKAP^135–225^ (mean±s.e.m., *n*=3). Scale bar, 3 μm.

Several microtubule-binding proteins interact with the negatively charged C-terminal regions of α- and β-tubulin, known as E-hooks^26^. The microtubule-binding region of SKAP, SKAP^135–225^, contains numerous conserved positively charged residues, several of which are positioned in the region comprised between residues 135 and 178 (Fig. 2a). In agreement with the hypothesis that electrostatic interactions mediate the binding of SKAP^135–225^ to microtubules, SKAP^135–225^ showed strongly reduced binding to microtubules that had been pretreated with the protease Subtilisin, which selectively removes the E-hooks (Fig. 2d).

To dissect the binding mechanism of SKAP^135–225^ for microtubules in detail, we resorted to chemical cross-linking with the bi-functional reagents BS2G (bis(sulfo-succinimidyl)glutarate) and DSS (di(succinimidyl)suberate), which cross-link the primary amines of lysine side chains within a distance compatible with the length of the cross-linkers (7.7 and 11.4 Å, respectively), followed by protease digestion and mass spectrometry (MS) analysis^27^. This approach identified several cross-links between lysine residues in SKAP^135–225^ and in α- and β-tubulin (Fig. 2b,c; Supplementary Data 1 and 2).

SKAP^135–225^ established cross-links with 10 distinct tubulin residues, including nine residues on α-tubulin (K60, K96, K112, K163, K311, K336, K370, K394 and K401) and one on β-tubulin (K252), which (with the exception of K60 and K370) show high cross-linking occurrence in MS analysis. These residues are positioned at the intra- and inter-tubulin dimer interface. Conversely, the two residues cross-linking with lower occurrence (K60 and K370) lie on the surface of α-tubulin. This distribution of lysine residues on α- and β-tubulin suggests that SKAP may recognize the interface of α- and β-tubulin.

The main microtubule receptor in the outer kinetochore, the Ndc80 complex, has also been shown to bind at the interface of α- and β-tubulin^28^,^29^, predicting that SKAP and Ndc80 might have at least in part overlapping binding sites on microtubules. This prediction was confirmed in a co-sedimentation competition experiment. When added at increasing concentration to a fixed concentration of Ndc80 complex and microtubules (both at 1 μM), SKAP^135–225^ progressively displaced Ndc80 and became enriched in the pellet fractions (Fig. 2e). To strengthen the notion that SKAP and Ndc80 bind overlapping sites on microtubules, a competition assay was also performed in a flow cell with fluorescent microtubules and at lower protein concentrations (100 nM tubulin (in microtubules stabilized with Taxol), 35 nM green fluorescent protein (GFP)-tagged Ndc80 complex and 0–4 μM mCherry-SKAP^135–225^). mCherry-SKAP^135–225^ displaced Ndc80 in a concentration-dependent manner, in agreement with the results of the co-sedimentation experiments. Thus, SKAP and Ndc80 have at least partly overlapping binding sites on microtubules (Fig. 2f).

### Identification of microtubule-binding residues in SKAP

**Figure 3:**
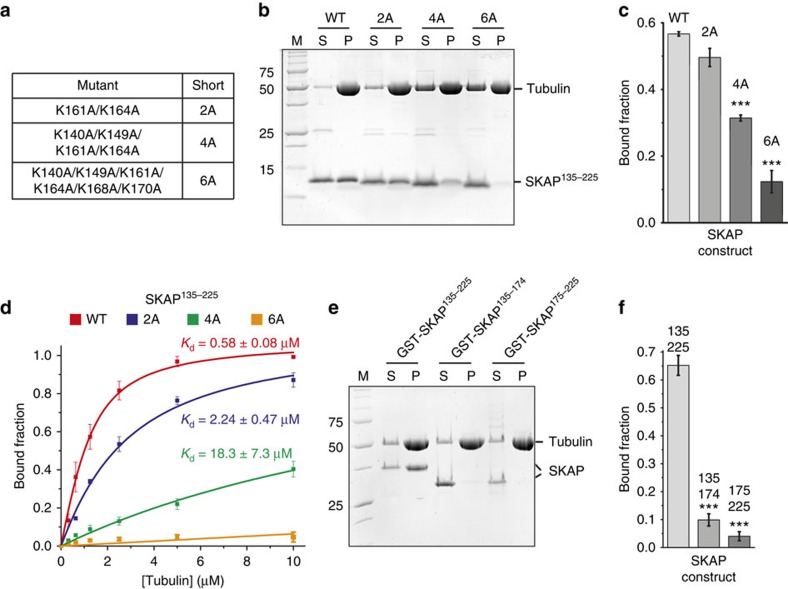
Multiple positively charged residues are required for microtubule binding of SKAP *in vitro*. (**a**) Summary of alanine point mutants created based on cross-linking analysis. (**b**) Representative SDS–PAGE of microtubule co-sedimentation assays with 3 μM taxol-stabilized microtubules and 3 μM SKAP^135–225^ wild type (WT) or indicated mutants. M, molecular weight marker; P, pellet fraction; S, soluble fraction. (**c**) Quantification of microtubule co-sedimentation assays shown in **b** (mean±s.e.m., *n*=3, *t*-test: ***P<0.001). (**d**) Quantification and fitting analysis of microtubule co-sedimentation assays with 0–10 μM taxol-stabilized microtubules and 1 μM SKAP^135–225^ WT or indicated mutants (mean±s.e.m., *n*=3). (**e**) Representative SDS–PAGE of microtubule co-sedimentation assays with 3 μM taxol-stabilized microtubules and 3 μM GST-SKAP^135–225^, GST-SKAP^135–174^ or GST-SKAP^175–225^. M, molecular weight marker; P, pellet fraction; S, soluble fraction. (**f**) Quantification of microtubule co-sedimentation assays shown in **e** (mean±s.e.m., *n*=3, *t*-test: ***P<0.001).

Our cross-linking analysis identified several lysine (K) residues in SKAP for being involved in microtubule binding, including K140, K149, K161, K164, K168 and K170. We created multiple alanine point mutants of these residues (Fig. 3a) and tested their effects on microtubule co-sedimentation binding assays. While the combination of the K161A and K164A mutations (‘2A' mutant) produced only an approximately fourfold reduction of microtubule-binding affinity, mutants exposing K140A, K149A, K161A and K164A (‘4A' mutant) or K140A, K149A, K161A, K164A, K168A and K170A (‘6A' mutant) were severely impaired (∼31-fold reduction of microtubule-binding affinity) or unable to bind microtubules, respectively (Fig. 3b–d).

Because all lysine residues identified by our cross-linking and mutational analysis lie within the N-terminal region of the SKAP^135–225^ segment, we asked if a shorter SKAP construct, SKAP^135–174^, was sufficient for tight microtubule binding. This was not the case, as SKAP^135–174^ was almost completely unable to bind microtubules. A complementary segment, SKAP^175–225^, was also unable to interact with microtubules (Fig. 3e,f). These observations suggest that the microtubule-binding site extends to the entire SKAP^135–225^ segment.

### SKAP binds to tubulin in solution

**Figure 4:**
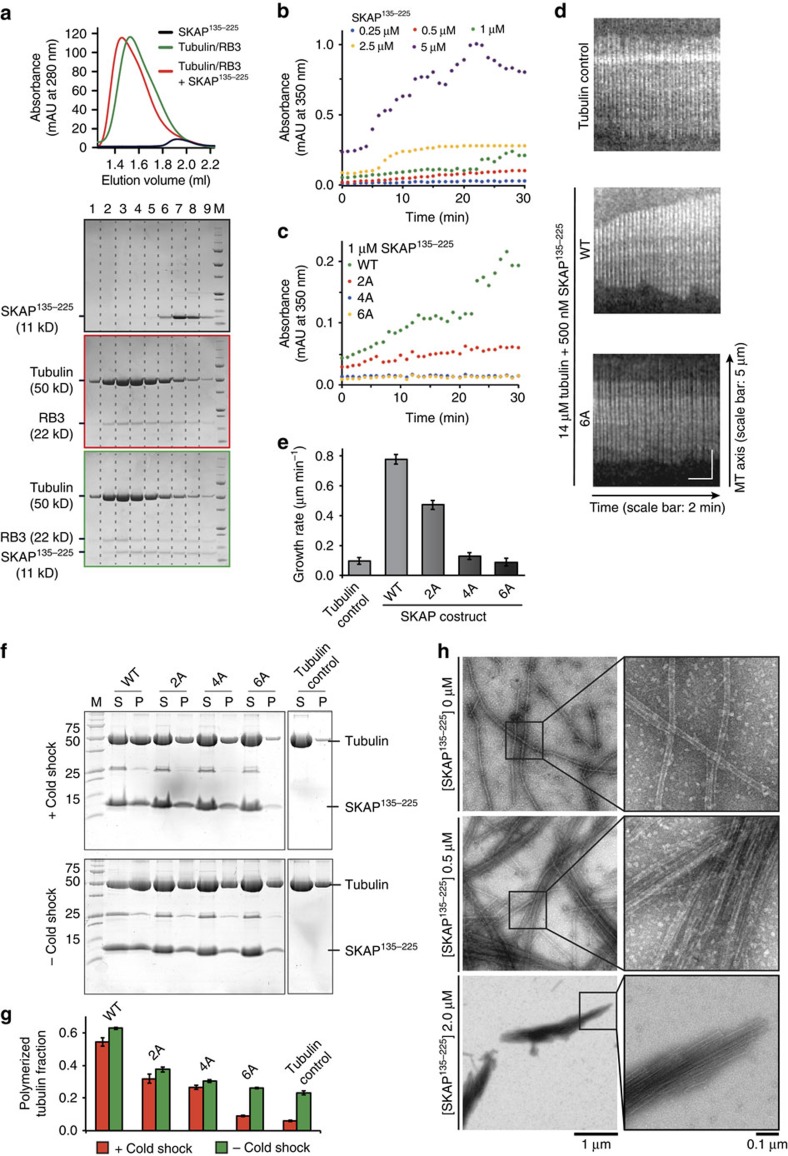
SKAP^135–225^ binds to tubulin in solution and promotes its polymerization and bundling *in vitro*. (**a**) Representative chromatogram and SDS–PAGE of analytical size-exclusion migration shift assays with SKAP^135–225^ and tubulin/RB3 complex on a Superdex 200 5/150. (**b**) *In vitro* Tubulin polymerization assays with 5 μM tubulin and 0.25-5 μM SKAP^135–225^ (*n*=4). Tubulin polymerization was followed via absorbance at 350 nm. (**c**) *In vitro* tubulin polymerization assays with 5 μM tubulin and 1 μM SKAP^135–225^ wild type (WT) or indicated mutants (*n*≥3). Tubulin polymerization was followed via absorbance at 350 nm. (**d**) Kymographs of microtubules observed in TIRF experiments in absence or presence of 500 nM SKAP^135–225^ WT or 6A mutant. Additional kymographs for tubulin control, WT and mutants are shown in Supplementary Fig. 4A. (**e**) Microtubule growth rates observed in TIRF experiments in absence or presence of 500 nM SKAP^135–225^ WT or indicated mutants (mean±s.e.m., *n*=3, for each condition ≥15 microtubules were analysed). (**f**) Representative SDS–PAGE of microtubule cold shock assay (top) and control without cold treatment (bottom) in absence or presence of SKAP^135–225^ WT or indicated mutants. M, molecular weight marker; P, pellet fraction; S, soluble fraction. Images originate from two different gels each as indicated by dividing lines. (**g**) Quantification of microtubule cold shock assay shown in **f** (mean±s.e.m., *n*=3). (**h**) Representative electron micrographs of negative-stained taxol-stabilized microtubules in the presence of 0-2 μM SKAP^135–225^.

Next, we investigated if the microtubule-binding region of SKAP can also mediate the interaction with tubulin in solution. To this end, we utilized a segment of the Stathmin-like protein RB3 to prevent tubulin from forming microtubules^30^. We then used size-exclusion chromatography, which separates based on size and shape, to obtain elution profiles of SKAP^135–225^, of the tubulin-RB3 complex, or of their combination (Fig. 4a). In AUC and cross-linking experiments, SKAP^135–225^ behaved as a trimer (Supplementary Fig. 3). Incidentally, because label-free MS analysis of the SKAP:Astrin complex predicts a 2:1 stoichiometry^12^, the overall stoichiometry of the SKAP:Astrin complex might be expected to be 6:2. The size-exclusion chromatography elution profile of SKAP^135–225^ was completely altered when the protein was mixed stoichiometrically with the tubulin:RB3 complex, with which it co-eluted (Fig. 4a). Thus, SKAP^135–225^ can interact not only with microtubules, but also with tubulin in solution.

This observation prompted us to ask if SKAP may nucleate the polymerization of microtubules at a concentration of tubulin that allows microtubule growth but not nucleation. In agreement with the hypothesis, SKAP^135–225^ induced microtubule polymerization in an established assay measuring light absorbance at 350 nm. This effect was concentration-dependent, with the largest effects obtained at a SKAP:tubulin concentration ratio approaching unity (Fig. 4b). The 2A, 4A and 6A SKAP mutants discussed above were severely impaired in this assay, with the 4A and 6A mutants being essentially unable to facilitate the nucleation of microtubules (Fig. 4c). Thus, SKAP may facilitate the incorporation of α/β-tubulin dimers in the microtubule lattice in a non-catalytic manner, and in a way that crucially depends on the direct interaction of SKAP with the tubulin dimer.

In total internal reflection fluorescence (TIRF) microscopy experiments, under conditions balancing the ratio of polymerization and depolymerization of microtubules in real time (Supplementary Fig. 4A), we observed that SKAP^135–225^ shifted the balance towards microtubule polymerization, due to a strong increase in the microtubule growth rate. The stimulation of microtubule growth rate was completely eliminated by mutations in the microtubule-binding domain of SKAP (Fig. 4d,e).

In the TIRF experiments, we also observed formation of microtubule bundles in the presence of SKAP^135–225^, suggesting that SKAP may bundle microtubules (Supplementary Fig. 4B). In control experiments, cold treatment caused depolymerization of microtubules (Fig. 4f,g). SKAP^135–225^ strongly suppressed microtubule depolymerization, but this stabilizing effect of SKAP was lost with the 6A mutant, and significantly suppressed with the 2A and 4A mutants. Notably, in the absence of a cold shock, the fraction of polymerized tubulin was greater in the presence of wild-type SKAP^135–225^ than in the presence of the mutants or in the tubulin control, indicating that SKAP may promote tubulin polymerization. Negative stain electron microscopy of microtubules in presence of SKAP^135–225^ revealed large microtubule bundles that were not observed in the absence of SKAP^135–225^ (Fig. 4h and Supplementary Fig. 5), further confirming the bundling effect of SKAP on microtubules. We speculate that this bundling effect is caused by the observed oligomerization properties of SKAP^135–225^ (Supplementary Fig. 3).

### Phenotype of microtubule-binding and SXIP mutants of SKAP

**Figure 5:**
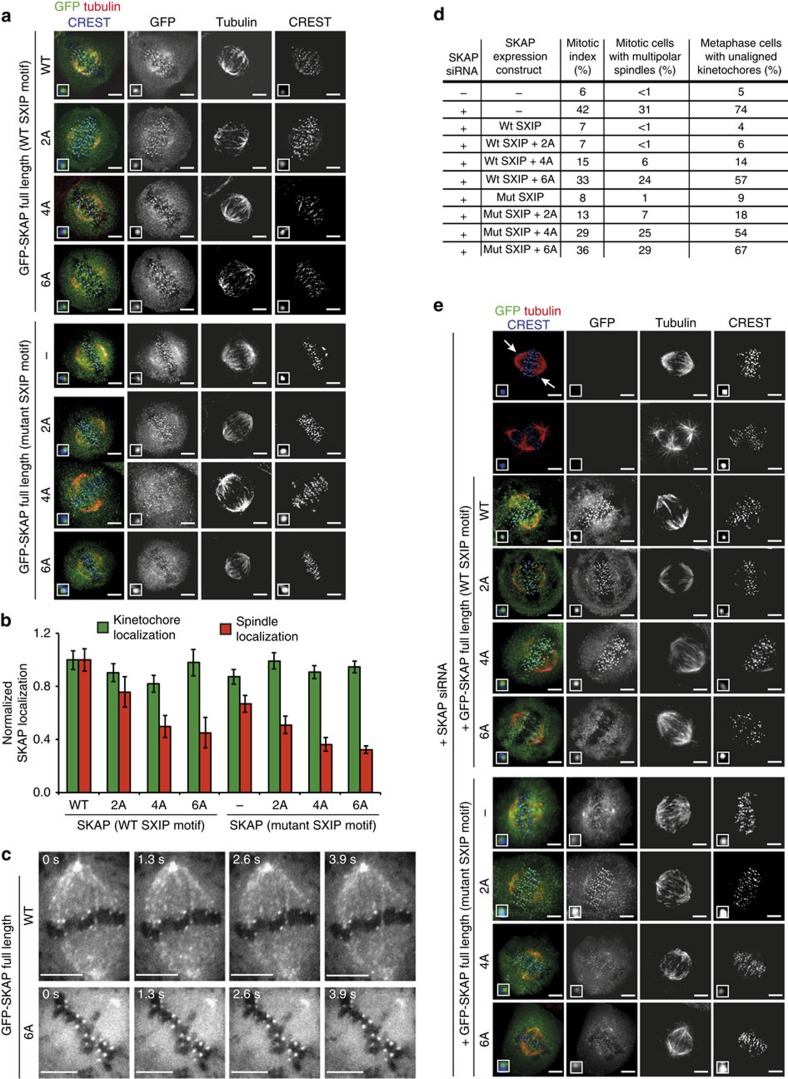
Multiple positively charged residues are required for microtubule binding and tip-tracking as well as function of SKAP *in vivo*. **a**) Representative immunofluorescence images of stable Flp-In T-REx cell lines expressing GFP-SKAP full-length wild type (WT), indicated microtubule-binding mutants (2A, 4A and 6A) or SXIP mutants (serine-leucine-leucine-proline (SLLP) mutated to serine=leucine-asparagine-asparagine (SLNN)). Cells were treated with doxycycline for 24 h and MG132 for 1.5-2 h before fixation. Scale bar, 5 μm. (**b**) Quantification of normalized GFP-SKAP fluorescence at the kinetochores or the spindle microtubules for immunofluorescence images shown in **a** (mean±s.e.m., *n*_kinetochores_≥110, *n*_spindles_≥10). (**c**) Live cell imaging of stable Flp-In T-REx cell lines expressing GFP-SKAP full-length WT or 6A mutant. The entire set of experiments, including the 2A and 4A mutants, is shown in Supplementary Fig. 6B Cells were treated with doxycycline for 24 h before imaging. Scale bar, 5 μm. (**d**) Summary of observed phenotypes in RNA interference (RNAi) and rescue experiments shown in **e** (*n*≥200). (**e**) Representative immunofluorescence images of RNAi and rescue experiments with stable Flp-In T-REx cell lines expressing GFP-SKAP full-length WT or indicated mutants. Scale bar, 5 μm.

To examine the localization of SKAP, we fused the full-length coding sequence of wild-type SKAP, and of the 2A, 4A and 6A mutants, to that of GFP. We then established stable inducible cell lines in which the levels of expression of wild type and mutant SKAP sequences after Doxycycline induction were approximately equal (Supplementary Fig. 6). In metaphase cells, wild-type GFP-SKAP localized to kinetochores and to the mitotic spindle (Fig. 5a), as previously observed^6^,^11^,^12^. We also analysed the localization of the microtubule-binding mutants of SKAP previously analysed in Fig. 3a. Kinetochore localization of all mutants was essentially unaltered. Conversely, the 4- and 6-alanine point mutants displayed strongly reduced spindle localization (Fig. 5a,b). These negative effects on spindle localization were compounded by addition of a mutation in the SXIP motif of SKAP. The latter, however, retained apparently normal kinetochore localization (Fig. 5a,b).

Wild-type GFP-SKAP tracked the tips of microtubules (Fig. 5c, Supplementary Fig. 6B and Supplementary Movie 1), in agreement with previous studies^17^,^18^. Conversely, the 6A mutant completely lost the ability to associate with microtubule plus ends, despite an intact SXIP motif (Fig. 5c, Supplementary Fig. 6B and Supplementary Movies 2, 3, 4, 5, which also document the behaviour of the 2A and 4A mutants). Thus, residues in the microtubule-binding domain of SKAP, in addition to the SXIP motif, are required for plus-end tracking. Both the kinetochore and pole localization of the 6A mutant appeared unaltered in these live cell experiments, suggesting that recruitment of SKAP:Astrin to these structures is independent of microtubules. Furthermore, because Astrin is required for kinetochore recruitment of SKAP (refs 11, 12), these results imply that the SKAP 6A mutant interacts with Astrin normally.

In cells in which SKAP had been depleted by RNA interference (Supplementary Fig. 7), we observed a number of spindle and chromosome alignment problems, including a dramatic increase in the occurrence of multipolar spindles, and the presence of pseudo-metaphases with multiple misaligned chromosomes, and a concomitant increase of the mitotic index, indicative of spindle assembly checkpoint activation^31^ (Fig. 5d; a gallery of these effects is shown in Fig. 5e). Re-expression of wild-type SKAP or the 2A mutant rescued these detrimental effects of SKAP depletion, while the 4A and 6A mutants rescued partly or not at all, respectively. Because the 6A mutant localizes normally to kinetochores (see above), these results imply that kinetochore localization in the absence of microtubule binding is insufficient to recapitulate the mitotic functions of SKAP. In line with the localization experiments in Fig. 5a,b, the effects of mutations in the microtubule-binding domain of SKAP were compounded by mutations in the SXIP motif (Fig. 5d,e).

### Kinetochore recruitment of SKAP:Astrin

Aurora B kinase has been shown to counter the localization of SKAP:Astrin to kinetochores^11^,^13^. Treatment with the small-molecule Aurora B inhibitor Hesperadin^32^ of metaphase-arrested HeLa cells, whose kinetochores are intensely decorated with endogenous SKAP, led to a small but significant increase in the levels of SKAP (Supplementary Fig. 8A,B). In cells treated with the microtubule-depolymerizing agent nocodazole, conversely, there was virtually no SKAP on kinetochores, but in this case addition of Hesperadin led to a large increase in SKAP at kinetochores (Supplementary Fig. 8A,B). Localization of SKAP to kinetochores in the absence of microtubules clearly indicates that microtubules may enhance, but are not required for, loading SKAP on kinetochores.

The KMN network (a 10-subunit super-complex of the Knl1, Mis12 and Ndc80 sub-complexes, already discussed in a previous paragraph) is considered the principal microtubule-binding site at the kinetochore^22^. Localization of the SKAP:Astrin complex to kinetochores also depends on the KMN subunits^13^,^18^. However, the mechanism of kinetochore recruitment of SKAP:Astrin is elusive. Mutants inactivating the SXIP motif in the N-terminal half of SKAP displayed normal kinetochore localization^17^, and two previous studies demonstrated that C-terminal segments of SKAP, roughly corresponding to the Astrin-binding domain^12^,^18^, are compatible with robust SKAP recruitment.

In microscale thermophoresis (MST), binding events are analysed by detecting changes in the diffusion of a (fluorescent) protein in a thermal gradient when varying concentrations of a second macromolecule are added^33^. By MST, we monitored the migration of mCherry-labelled SKAP^159–316^ in the presence of progressively higher concentrations of recombinant Mis12 or Ndc80 complexes. These experiments indicated that SKAP^135–225^ binds the Mis12 and Ndc80 complexes with *K*_d_s of 65 nM and 56 nM, respectively (Supplementary Fig. 8C,D). However, we were unable to detect binding of SKAP^135–225^ to the Mis12 or Ndc80 complexes with other methods, including size-exclusion chromatography and solid phase pull-down assays, which is surprising given the remarkably low *K*_d_s (that is, high affinities) emerging from the MST experiments. Equally puzzling, MST failed to detect an interaction between the Mis12 and Ndc80 complexes (Supplementary Fig. 8E), which we have consistently detected by an array of techniques, including size-exclusion chromatography, solid phase binding and isothermal titration calorimetry. By isothermal titration calorimetry, we determined that the interaction of the Mis12 and Ndc80 complexes has a *K*_d_ as low as ∼10 nM (refs 34, 35), and it is surprising that MST failed to detect it. Collectively, these observations call for caution in the interpretation of binding data, especially when they are obtained with a single approach.

Thus, whether SKAP^135–225^ binds the Mis12 and Ndc80 complexes directly will require further investigations. The SKAP-binding domain of Astrin is not sufficient for kinetochore localization, because the C-terminal coiled-coil region of Astrin is also required^12^. Furthermore, depletion of Astrin prevents kinetochore recruitment of SKAP (refs 11, 12). Thus, kinetochore binding by the SKAP:Astrin complex may require a composite surface including segments from both SKAP and Astrin.

## Discussion

The SKAP:Astrin protein assembly is large and structurally and functionally complex. A previous report identified conditions for refolding of the full-length Astrin sequence^8^, but our attempts to obtain full-length Astrin using a similar approach were unsuccessful. Here, we have carried out the first thorough *in vitro* analysis of the SKAP:Astrin complex, focusing especially on the inter-subunit interaction and on a minimal microtubule-binding domain (SKAP^135–225^). More extensive functional analyses of the SKAP:Astrin complex *in vitro* will require obtaining stable soluble versions of larger segments of the two proteins, a goal that has been difficult to achieve in this study given the poor solubility of most Astrin constructs.

We mapped the binding site of SKAP^135–225^ on microtubules, and determined that it coincides, at least in part, with the binding site of the Ndc80 complex. We also identified a number of positively charged residues on SKAP^135–225^ that are important for microtubule binding, as shown by a point mutation analysis *in vitro* and in cells. Our studies demonstrate that SKAP contains a *bona fide* microtubule-binding domain, whose integrity is required for rescuing the significant structural spindle malformations and the congression defects caused by depletion of SKAP.

The microtubule-binding domain we have identified likely cooperates with the SXIP motif that targets the SKAP:Astrin complex to growing plus ends of microtubules^12^,^17^,^18^. There are other examples of proteins that combine a SXIP motif with a functional microtubule-binding domain, such as mitotic centromere-associated kinesin (MCAK) (ref. 20). A possible functional significance of this combination of motifs is that a specific function of the microtubule-binding domain is delivered specifically or preferentially to the microtubule plus ends. *In vitro*, the microtubule-binding domain of SKAP appears to favour microtubule growth (this study), and it is possible that this is the crucial activity of the SKAP:Astrin complex that needs to be delivered to plus ends of microtubules and to kinetochores.

In addition to the SXIP motif and to the microtubule-binding domain we described here, a C-terminal segment of Astrin (residues 955–1,193) was also found to associate with microtubules *in vitro*^11^. Using a very similar construct of Astrin (residues 966–1,175), however, we were unable to detect microtubule binding. We also note that a construct encompassing the Astrin C-terminal segment (but not the SKAP-binding region) is unable to support spindle localization^12^, while a segment lacking this region retains spindle localization^7^.

During maturation from the initial lateral attachments to the final end-on attachments, kinetochores likely transit through distinct physical and chemical states^1^. A number of proteins that are initially present on kinetochores before microtubule attachment, most notably the Rod-Zwilch-ZW10 complex and its associated proteins Spindly and Dynein-Dynactin, become released from kinetochores on initial attachment^1^,^31^. Microtubule depolymerases, such as MCAK and Kif2b, are also released in the passage from lateral to end-on attachment^13^. Other proteins, including SKAP:Astrin and the SKA (spindle and kinetochore associated) complex, are only recruited robustly to attached kinetochores, and appear to have a function in the final stabilization of end-on attachment that precedes entry into anaphase^1^. Aurora B kinase appears to control this pattern, because it contributes to the recruitment of early kinetochore binders such as RZZ and MCAK, and suppresses the recruitment of the late kinetochore binders until its own activity attenuates, probably as a result of separation from its substrates^36^. This behaviour likely subtends to the well know role of Aurora B in the correction of erroneous kinetochore–microtubule attachments and in the establishment of chromosome bi-orientation^15^.

## Methods

### Expression and purification of recombinant proteins

Genes encoding SKAP^135–225^, SKAP^1–158^, SKAP^159–225^, SKAP^226–316^, SKAP^159–316^, Astrin^1–239^, Astrin^1–481^ and Astrin^966–1,175^ constructs were PCR amplified from a human cDNA library (primer data are collected in Supplementary Table 1) and subcloned into a pGEX-6p-2rbs vector, a modified version of the pGEX-6p vector (GE Healthcare, Piscataway, NJ), with an N-terminal 3C-cleavable GST-tag. SKAP mutants were generated using site-directed mutagenesis. All plasmids were verified by DNA sequencing. Proteins were expressed in *Escherichia coli* OverExpress C41 (DE3; Lucigen, Middleton, WI) for ∼16 h at 18 °C after induction with 0.1 mM isopropyl-β-D-thiogalactoside. Cells were lysed by sonication in buffer containing 50 mM Hepes, pH 7.5, 300 mM NaCl, 10% (v/v) glycerol, 5 mM dithioerythritol (DTE), 0.1% Tween-20 and protease inhibitor cocktail (Serva, Heidelberg, Germany). The cleared lysates were purified by affinity chromatography on GSTrap FF columns (GE Healthcare). In case of SKAP^159–225^, SKAP^226–316^, SKAP^159–316^, Astrin^1–239^, Astrin^1–481^ and Astrin^966–1,175^, cleavage of the GST-tag was performed overnight at 4 °C on the column. For SKAP^1–158^, the GST-tag was not cleaved. Subsequently, proteins were purified by anion exchange chromatography on HiTrap Q FF columns (GE Healthcare) and size-exclusion chromatography on a Superdex 200 16/60 (GE Healthcare) in 20 mM Hepes pH 7.5, 300 mM NaCl, 10% (v/v) glycerol and 2 mM DTE.

For the SKAP^159–316^:Astrin^482–850^ complex, the PCR amplified genes were subcloned into the two multiple cloning sites of a MultiBac pFL-derived vector^37^ with a C-terminal His_6_-tag on Astrin^482–850^. Baculovirus was produced in accordance to ref. 37. Specifically, bacmid was generated by recombination in DH10EMBacY cells for 5 h at 37 °C and successful transposition was selected via blue/white screening. Isolated bacmid was used to transfect *Spodoptera frugiperda* Sf9 cells (Invitrogen) in Sf-900 III serum-free medium (Invitrogen) at 27 °C for 3 days. Subsequently, transfected cells were incubated at 27 °C for 4 days in fresh medium containing 1 × 10^6^ Sf9 cells per ml. Cells were centrifuged at 1,000*g* for 5 min and the supernatant was used for further virus amplification in Sf9 cells with a ratio of 1:100 at 27 °C for 4 days. The SKAP^159–316^:Astrin^482–850^ complex was expressed for 4 days at 27 °C in *Tnao*38 cells using a virus:culture ratio of 1:100. Cells were lysed in buffer containing 50 mM Hepes, pH 7.5, 300 mM NaCl, 10% (v/v) glycerol, 20 mM imidazole, 5 mM β-mercaptoethanol, 0.5% Tween-20 and protease inhibitor cocktail (Serva). The protein complex was purified by affinity chromatography on a HisTrap FF column (GE Healthcare) and subsequent size-exclusion chromatography on a Superose 6 10/300 (GE Healthcare) in 20 mM Hepes pH 7.5, 300 mM NaCl, 10% (v/v) glycerol and 2 mM DTE. Ndc80 and Mis12 complexes were expressed and purified as also described elsewhere^34^. Specifically, Mis12 complex was expressed in *E. coli* BL21(DE3)pLysS cells in Terrific broth at 18 °C overnight after induction with 0.1 mM isopropyl-β-D-thiogalactoside. Cells were harvested by centrifugation, resuspended and lysed by sonication. The cleared lysate was purified via affinity chromatography on Ni-NTA agarose beads (QIAGEN), followed by ion-exchange chromatography on a ResourceQ column and subsequent size-exclusion chromatography on a Superdex 200 10/300 column (GE Healthcare). Insect cells expressing the Ndc80 complex were harvested by centrifugation, resuspended and lysed by sonication. The cleared lysate was purified via affinity chromatography on Ni-NTA agarose beads (QIAGEN) and subsequent size-exclusion chromatography on a Superose 6 10/300 column (GE Healthcare).

### Microtubule co-sedimentation assays

Tubulin was purchased from Cytoskeleton, Inc. (Denver, CO) and was polymerized according to manufacturer's instructions. For Subtilisin-treated microtubules, 6 μM taxol-stabilized microtubules were incubated with 100 μg ml^−1^ Subtilisin A for 45 min at 30 °C. The reaction was quenched by adding 10 mM phenylmethylsulfonyl fluoride (PMSF). Digested microtubules were pelleted and resuspended in BRB80 buffer. Microtubules and proteins were mixed in a final volume of 35 μl in 25 mM Hepes, pH 7.5, 150 mM NaCl, 1 mM MgCl_2_, 1 mM EGTA and 2 mM DTE. For microtubule-binding reactions, 3 μM microtubules and 3 μM protein of interest were used. In case of *K*_d_ calculations, 0-10 μM taxol-stabilized microtubules (tubulin dimer concentration), 1 μM BSA and 1 μM protein of interest (protein monomer concentration) were mixed. Reaction mixtures were incubated for 10 min at room temperature, transferred onto 100 μl of cushion buffer (25 mM Hepes, pH 7.5, 150 mM NaCl, 1 mM MgCl_2_, 1 mM EGTA, 50% glycerol and 50 μM taxol) and ultracentrifuged at 350,000 *g* for 10 min at 25 °C. Supernatants and pellets were analysed by SDS–polyacrylamide gel electrophoresis (SDS–PAGE). Quantification was done as also described elsewhere^29^. In brief, gel densitometry was carried out with ImageJ 1.49 (NIH). Bound fractions were obtained by dividing values of the pellet fraction by the sum of pellet and supernatant. Normalized binding data were fitted using Origin7.0 (OriginLab, Northampton, MA) to the following quadratic equation:


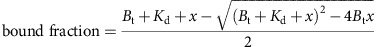


*B*_t_ is the maximal fractional protein-tubulin complex, *K*_d_ is the dissociation constant and *x* is the concentration of tubulin dimer.

### Chemical cross-linking and mass spectrometric analysis

10 μM SKAP^135–225^ and 10 μM taxol-stabilized microtubules were mixed with DSS-H12/D12 (Creative Molecules, www.creativemolecules.com) in a weight ratio of 1:2.5 or with BS2G-H6/D6 (Creative Molecules) in a weight ratio of 1:1 in a final volume of 150 μl. After incubation for 30 min at 37 °C the reaction was quenched by adding 100 mM ammonium bicarbonate and incubating 15 min at 37 °C. Cross-linked proteins were digested and the peptides were enriched and analysed by liquid chromatography coupled to tandem mass spectrometry using a hybrid LTQ-Orbitrap Elite instrument (Thermo Fisher Scientific, Waltham, MA)^27^. Cross-links were identified by the dedicated software xQuest (ref. 38). False discovery rates were estimated using xProphet (ref. 38) and results were filtered according to the following parameters: false discovery rates=0.05, min delta score=0.90, MS1 tolerance window of −4 to 4 p.p.m., Id-score >22. Cross-links were visualized using the xVis web server^39^.

### Fluorescence microtubule flow cell assays

Coverslips and glass slides were cleaned by sonication in isopropanol and 1 M KOH or 1% Hellmanex and 70% ethanol, respectively. After functionalization of coverslips with 5% biotinylated poly-L-lysine-PEG for 30 min, flow cells were created with a volume of 10–15 μl. Flow cells were passivated with 1% pluronic F-127 for 1 h and coated with avidin for 30–45 min. After incubation with 100 nM microtubules (10% biotinylated, 10% fluorescent HiLyte-647 labelled, Cytoskeleton, Inc., polymerized according to manufacturer's instructions) for 10-20 min, 35 nM GFP-tagged Ndc80 complex was added in presence of mCherry-SKAP^135–225^ at the indicated concentration. Flow cells were sealed with wax and imaged with spinning disk confocal microscopy on a 3i Marianas system (Intelligent Imaging Innovations, Göttingen, Germany) equipped with Axio Observer Z1 microscope (Zeiss, Oberkochen, Germany), a CSU-X1 confocal scanner unit (Yokogawa Electric Corporation, Tokyo, Japan), Plan-Apochromat × 100/1.4 numerical aperture (NA) differential interference contrast (DIC) oil objective (Zeiss), Orca Flash 4.0 sCMOS Camera (Hamamatsu, Hamamatsu City, Japan) and controlled by Slidebook Software 6.0 (Intelligent Imaging Innovations). Images were acquired as z-sections at 0.27 μm and maximal intensity projections were made with Slidebook Software 6.0 (Intelligent Imaging Innovations). Quantification was done with ImageJ 1.49 (NIH) and intensities were normalized to microtubule signals.

### Cell line generation and cell culture

Codon-optimized SKAP genes (Invitrogen, Carlsbad, CA) were PCR amplified and subcloned into a pCDNA5/FRT/TO-EGFP-IRES vector, a modified version^35^ of the pCDNA5/FRT/TO vector (Invitrogen). Mutations were generated using site-directed mutagenesis. All plasmids were verified by DNA sequencing. Flp-In T-REx HeLa cells used to generate stable doxycycline-inducible cell lines were a gift from S.S. Taylor (University of Manchester, Manchester, England, UK). Flp-In T-REx HeLa host cell lines were maintained in DMEM (PAN Biotech, Aidenbach, Germany) with 10% tetracycline-free FBS (Clontech, Shiga, Japan) supplemented with penicillin and streptomycin (GIBCO, Carlsbad, CA). Flp-In T-REx HeLa expression cell lines were generated as also described elsewhere^40^. Specifically, Flp-In T-Rex HeLa host cells were cotransfected with pOG44 and pCDNA5/FRT/TO expression plasmid in a ratio of 9:1 using FuGENE transfection agent. After 48 h, Flp-In T-Rex HeLa expression cell lines were put under selection for 2 weeks in DMEM with 10% tetracycline-free FBS supplemented with 250 μg ml^−1^ hygromycin and 4 μg ml^−1^ blasticidin (Invitrogen). Resulting colonies were pooled and maintained in DMEM with 10% tetracycline-free FBS supplemented with 250 μg ml^−1^ hygromycin and 4 μg ml^−1^ blasticidin (Invitrogen). HeLa cells were maintained in DMEM with 10% tetracycline-free FBS.

### Immunofluorescence

Flp-In T-REx HeLa cells were grown on coverslips precoated with poly-D-lysine (Millipore) and poly-L-lysine (Sigma-Aldrich, St Louis, MO). Gene expression was induced by addition of 0.1 μg ml^−1^ doxycycline (Sigma-Aldrich) for 24 h and cells were treated with 10 μM MG132 (Calbiochem, Darmstadt, Germany) for 2–3 h before fixation with 4% paraformaldehyde and permeabilization with 0.1% Triton X-100. For localization studies with endogenous SKAP, HeLa cells were grown on precoated coverslips and treated with 10 μM MG132 or 3.3 μM nocodazole for 3 h and 500 nM Hesperadin for 1.5 h before fixation and permeabilization.

Cells were stained for SKAP (rabbit polyclonal anti-SKAP^135–225^ antibody made in cooperation with Cogentech, Milan, Italy, 1:1,000), α-tubulin (mouse monoclonal anti-α-tubulin antibody, DM1A, Sigma-Aldrich code T9026, 1:500) and CREST/anti-centromere antibodies (Antibodies Inc., code 15-234-0001, Davis, CA, 1:100), diluted in 2% BSA-PBS for 1.5 h. As secondary antibodies donkey anti-rabbit Alexa Fluor 488 (Invitrogen, code A-21206), goat anti-human Alexa Fluor 647 (Invitrogen, code A-21445) and goat anti-mouse Rhodamine Red-X (Jackson ImmunoResearch Laboratories, Inc., code 115-295-003, West Grove, PA) were used. DNA was stained with 0.5 μg ml^−1^ 4,6-diamidino-2-phenylindole (Serva). Coverslips were mounted with Mowiol mounting media (Calbiochem). Cells were imaged at room temperature using a DeltaVision Elite imaging system (Applied Precision, Issaquah, WA) with an inverted Olympus IX71 microscope (Olympus, Shinjuku, Tokyo, Japan) equipped with a U Plan-Apochromat × 60/1.42NA oil objective (Olympus) and a CoolSNAP HQ2 camera (Photometrics, Tucson, AZ). Images were acquired as z-sections at 0.2 μm. Image deconvolution and maximal intensity projections were done with softWoRx 5.0 (Applied Precision). Quantification of kinetochore and spindle microtubule signals was performed with unmodified z-series images using the spot and the surface function of Imaris 7.3.4 software (Bitplane, Zurich, Switzerland). After background subtraction the GFP signals were normalized to those of the GFP-SKAP full-length cell line. For quantification of endogenous SKAP localization, intensities obtained for SKAP were normalized to CREST signals.

### RNA interference

For SKAP depletion, Flp-In T-REx HeLa cells were transfected with SKAP siRNA (5′-AGGCUACAAACCACUGAGUAA-3′) using HyPerFect transfection reagent (Qiagen) for 72 h. For rescue experiments, the expression of GFP fusion proteins in SKAP-depleted cells was induced for 24 h before fixation of the cells. Cells were stained and imaged as described above. Full blots in Supplementary Fig. 9.

### Tip-tracking experiments

Flp-In T-REx HeLa cells were grown in μ-Slide 8 Well ibiTreat chambers (ibidi, Martinsried, Germany) in CO_2_ independent medium (Thermo Fisher Scientific) with 10% tetracycline-free FBS supplemented with 250 μg ml^−1^ hygromycin and 4 μg ml^−1^ blasticidin (Invitrogen). Gene expression was induced by addition of 0.05 μg ml^−1^ doxycycline (Sigma-Aldrich) for 24 h. Live imaging of cells was done with spinning disk confocal microscopy on a 3i Marianas system (Intelligent Imaging Innovations) equipped with Axio Observer Z1 microscope (Zeiss), a CSU-X1 confocal scanner unit (Yokogawa Electric Corporation), Plan-Apochromat × 100/1.4 oil objective (Zeiss), Orca Flash 4.0 sCMOS Camera (Hamamatsu) and controlled by Slidebook Software 6.0 (Intelligent Imaging Innovations). Image series were acquired as z-sections at 0.27 μm with an interval of ∼1,300 ms for 90 time points and maximal intensity projections were made with Slidebook Software 6.0 (Intelligent Imaging Innovations).

### Analytical size-exclusion migration shift assay

Protein samples containing tubulin/RB3 complex, SKAP^135–225^ or their combination were prepared at a concentration of 15 μM in size-exclusion chromatography buffer (20 mM Hepes pH 7.5, 150 mM NaCl, 5% (v/v) glycerol and 2 mM DTE) in a final volume of 50 μl. After incubation for 1 h on ice samples were analysed via size-exclusion chromatography on a Superdex 200 5/150 GL (GE Healthcare). Protein containing fractions were analysed by SDS–PAGE.

### Tubulin polymerization assay

Tubulin polymerization was measured in a turbidity experiment by following the absorbance at 350 nm for 30 min at 37 °C. tubulin was incubated at 5 μM in presence of the indicated proteins in a final volume of 100 μl in 20 mM Hepes, pH 7.5, 150 mM NaCl, 5% glycerol, 4 mM MgCl_2_, 2.5 mM GTP and 2 mM DTE.

### TIRF microscopy

Coverslips and glass slides were prepared and functionalized as described for the fluorescence microtubule flow cell assays. After incubation with avidin the flow cells were washed with reaction buffer (25 mM Hepes, pH 7.5, 150 mM NaCl, 10% (v/v) glycerol, 1 mM MgCl_2_, 1 mM EGTA, 2 mM DTE) including an oxygen scavenger mix consisting of 4.5 mg ml glucose, 0.2 mg ml^−1^ glucose oxidase, 35 μg ml^−1^ catalase and 10 mM DTE. The chambers were incubated for 10–20 min with 20 μl of a microtubule seed mix containing 400 nM guanosine-5′ [(α,β)-methyleno]triphosphate (GMPCPP) microtubule seeds (15% biotinylated and 15% alexa-647 labelled; incubated for 30 min at 34 °C with 1 mM GMPCPP), oxygen scavenger mix, 0.5 mg ml casein and 0.1% (w/v) methylcellulose in reaction buffer. After washing with 50 μl of reaction buffer including oxygen scavenger mix a tubulin mix (14 μM Rhodamine labelled tubulin, 2.5 mM GTP, oxygen scavenger mix, 0.35 mg ml% casein and 0.1% (w/v) methylcellulose in reaction buffer) was added in the presence or absence of 500 nM SKAP wild type or mutants. The flow cells were sealed with wax and imaged at 25 °C with a VisiTIRF Fluorescence Imaging System (Visitron Systems GmbH, Puchheim, DE) with a Nikon Eclipse Ti-E inverted microscope (Nikon, Tokyo, JP), an Apo-TIRF × 100/1.49NA oil objective (Nikon) and an Evolve Delta camera (Photometrics). 200 time points were imaged every 5 s. Kymographs were made with ImageJ 1.49 (NIH). Kymographs and Supplementary Figures show the 561-nm channel, which collects signals from the free Rhodamine labelled tubulin. For calculation of growth rates, the microtubule growth between subsequent catastrophes was analysed for several events on at least 15 different microtubules. The increase in microtubule length, measured with ImageJ 1.49 (NIH), was divided by the time between the two catastrophes.

### Microtubule cold shock assay

Bovine α-/β-tubulin (10 μM; Cytoskeleton, Inc., Denver, US-CO) were incubated at 34 °C for 30 min either alone or in the presence of 10 μM protein of interest in a buffer containing 25 mM Hepes, pH 7.5, 150 mM NaCl, 5% (v/v) glycerol, 4 mM MgCl_2_, 1 mM EGTA, 2 mM DTE and 2.5 mM GTP. Afterwards, the samples were incubated for 10 min on ice and centrifuged on top of 100 μl cushion buffer (25 mM Hepes, pH 7.5, 150 mM NaCl, 1 mM MgCl_2_, 1 mM EGTA, 50% (v/v) glycerol) in a TLA 120.1 rotor at 350,000*g* for 10 min at 4 °C. As control, samples with the same composition were treated without cold shock and centrifuged at 350,000*g* for 10 min at 34 °C. Supernatants and pellets were analysed via SDS–PAGE and quantified with ImageJ 1.49 (NIH). The amount of polymerized tubulin was quantified by dividing the amount of tubulin in the pellet by its sum in supernatant and pellet.

### Microscale thermophoresis

Protein samples were prepared at room temperature as serial 1:1 dilutions with 150 nM mCherry-SKAP^159–316^ and 1.53-12,500 nM Mis12 complex or 0.76-6,250 nM Ndc80 complex, respectively, in 20 mM Hepes, pH 7.5, 150 mM NaCl, 5% (v/v) glycerol, 0.5 mg ml^−1^ BSA and 5 mM DTE. For Mis12 complex-Ndc80 complex interaction, 20 nM GFP-tagged Ndc80 complex were incubated with 0.1953–100 nM Mis12 complex in 10 mM Hepes, pH 7.5, 150 mM NaCl, 5% (v/v) glycerol, 0.05% (v/v) Triton X-100 and 5 mM DTT. After spinning for 5 min at 20,000*g* samples were transferred to standard glass capillaries (NanoTemper Technologies GmbH, Munich, Germany) and analysed with a Monolith NT.115 instrument (NanoTemper Technologies GmbH). The laser was switched on after 5 s for a duration of 30 s (MST-Power: 60, LED-Power: 90). Experiments were controlled with NT Control software (NanoTemper Technologies GmbH) and analysed with NT Analysis software (NanoTemper Technologies GmbH). Initial fluorescence and thermophoresis/temperature jump signals were normalized to signals of sample with lowest concentration of titrated unlabelled protein, plotted against the concentration of titrated unlabelled protein and fitted with Origin7.0 (OriginLab) using following equation (NanoTemper Technologies GmbH):


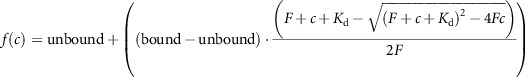


with *f*(*c*) as concentration-dependent signal, unbound as signal of unbound state, bound as signal of bound state, *F* as fluorophore concentration, *c* as concentration of titrated unlabelled protein and *K*_d_ as dissociation constant.

### Preparation of negative stain specimens and electron microscopy

Freshly prepared SKAP^159–316^:Astrin^482–850^ complex was diluted after size-exclusion chromatography and inspected by negative staining as described previously^41^. Briefly, 4 μl of the diluted sample were adsorbed for 40 s to a freshly glow-discharged carbon-coated 400 mesh copper grid (G2400C, Plano GmbH, Wetzlar, Germany). Excess solution was blotted off and specimens were washed three times with size-exclusion chromatography buffer and once with freshly prepared 0.75% uranyl formate (SPI Supplies/Structure Probe, West Chester, PA) before being stained with uranyl formate solution for 1 min. Excess staining solution was removed by blotting and the specimen was air-dried. Samples were imaged with a JEOL1400 microscope equipped with a LaB_6_ cathode operated at an acceleration voltage of 120 kV. Digital micrographs were recorded using a 4k × 4k CMOS camera F416 (TVIPS, Gauting, Germany) under minimal dose conditions.

To analyse the microtubule bundling effect of SKAP, 10 μM bovine tubulin (Cytoskeleton) was mixed with 0-2 μM SKAP^135–225^ in reaction buffer (25 mM Hepes, pH 7.5, 150 mM NaCl, 5% (v/v) glycerol, 4 mM MgCl_2_, 1 mM EGTA, 2 mM DTE and 2.5 mM GTP) and incubated 10 min at 34 °C. After addition of 50 μM taxol the samples were incubated for 30 min at 34 °C. Four microlitres of undiluted sample were adsorbed onto freshly glow-discharged, carbon-coated grids for 15 s and prepared and imaged as described above.

### Sedimentation velocity analytical ultracentrifugation

Sedimentation velocity experiments were performed in an Optima XL-A AUC (Beckman Coulter, Palo Alto, US-CA) with Epon charcoal-filled double-sector quartz cells and an An-60 Ti rotor (Beckman Coulter, Palo Alto, US-CA). Before the analysis samples were dialysed against buffer (20 mM Hepes, pH 7.5, 300 mM NaCl, 10% glycerol and 2 mM DTE) that was used as blank. Samples were centrifuged at 42,000 r.p.m. at 20 °C and 500 radial absorbance scans at 280 nm were collected with a time interval of 1 min. The data were analysed using the SEDFIT software^42^ in terms of continuous distribution function of sedimentation coefficients (c(S)). The protein partial specific volume was estimated from the amino acid sequence using the programme SEDNTERP. Data were plotted using the programme GUSSI.

## Additional information

**How to cite this article:** Friese, A. *et al*. Molecular requirements for the inter-subunit interaction and kinetochore recruitment of SKAP and Astrin. *Nat. Commun.* 7:11407 doi: 10.1038/ncomms11407 (2016).

## Supplementary Material

Supplementary Information Supplementary Figures 1-9 and Supplementary Table 1.

Supplementary Data 1 Excel datasheet containing list of cross-links between microtubules and SKAP obtained in the presence of the cross-linker BS2G

Supplementary Data 2 Excel datasheet containing list of cross-links between microtubules and SKAP obtained in the presence of the cross-linker DSS

Supplementary Movie 1 Movie documenting the mitotic localization of GFP-SKAP wild type

Supplementary Movie 2 Movie documenting the mitotic localization of GFP-SKAP-2A mutant (high expression level)

Supplementary Movie 3 Movie documenting the mitotic localization of GFP-SKAP-2A mutant (low expression level)

Supplementary Movie 4 Movie documenting the mitotic localization of GFP-SKAP-4A mutant

Supplementary Movie 5 Movie documenting the mitotic localization of GFP-SKAP-6A mutant
