# Supplementary material for: Molecular requirements for the inter-subunit interaction and kinetochore recruitment of SKAP and Astrin
Source: Nat Commun. 2016 Apr 20;7:11407. doi: 10.1038/ncomms11407 (PMC4843017; doi:10.1038/ncomms11407)
Supplement: Supplementary Information — Supplementary Figures 1-9 and Supplementary Table 1. [file ncomms11407-s1.pdf]

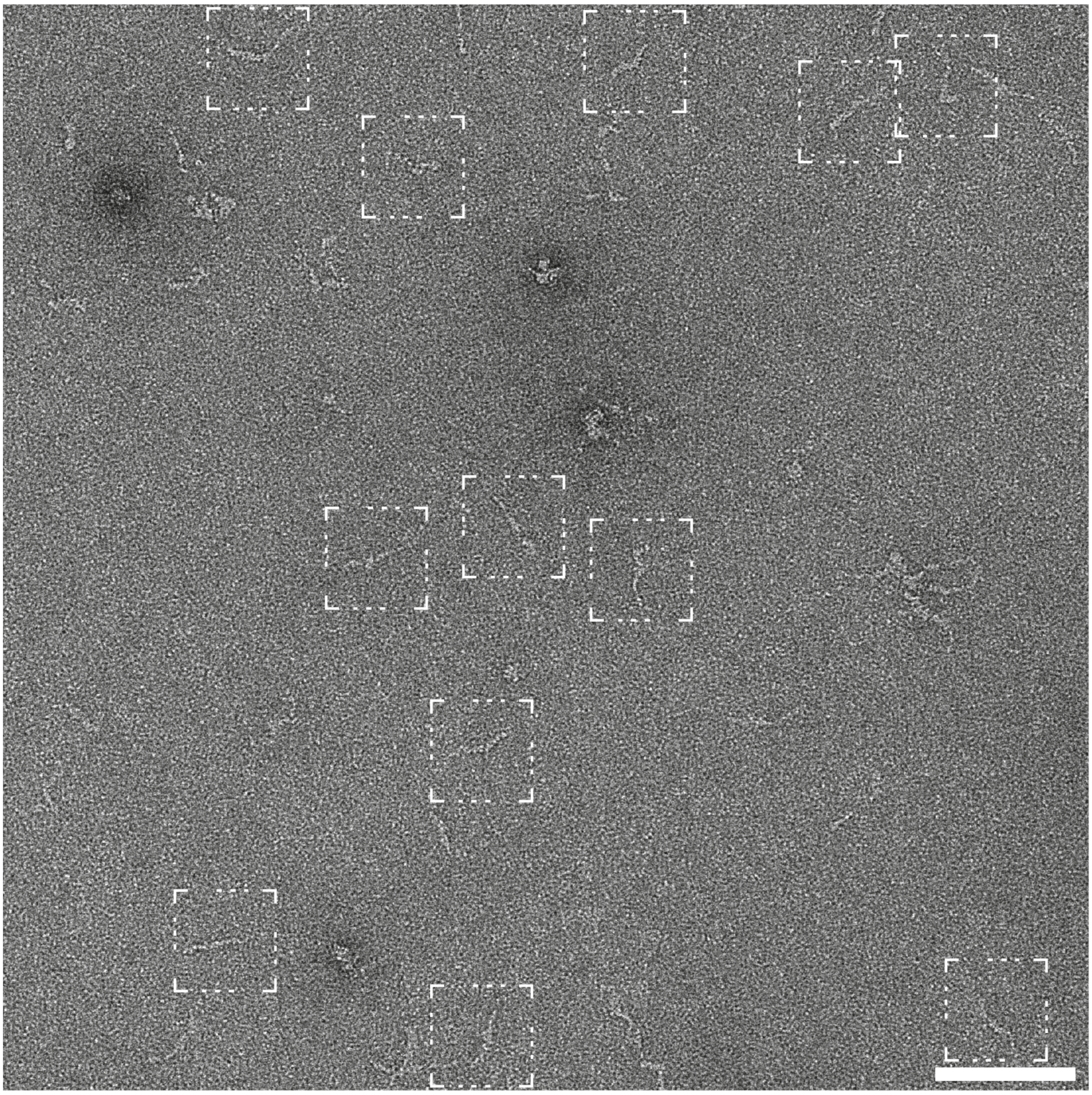

Supplementary Fig. 1

Supplementary Figure 1. The SKAP$^{159\text{-}316}$:Astrin$^{482\text{-}850}$ complex is highly elongated and flexible. Larger field of view for the negatively-stained SKAP$^{159\text{-}316}$:Astrin$^{482\text{-}850}$ complex shown in Figure 1C. Scale bar: 100 nm.

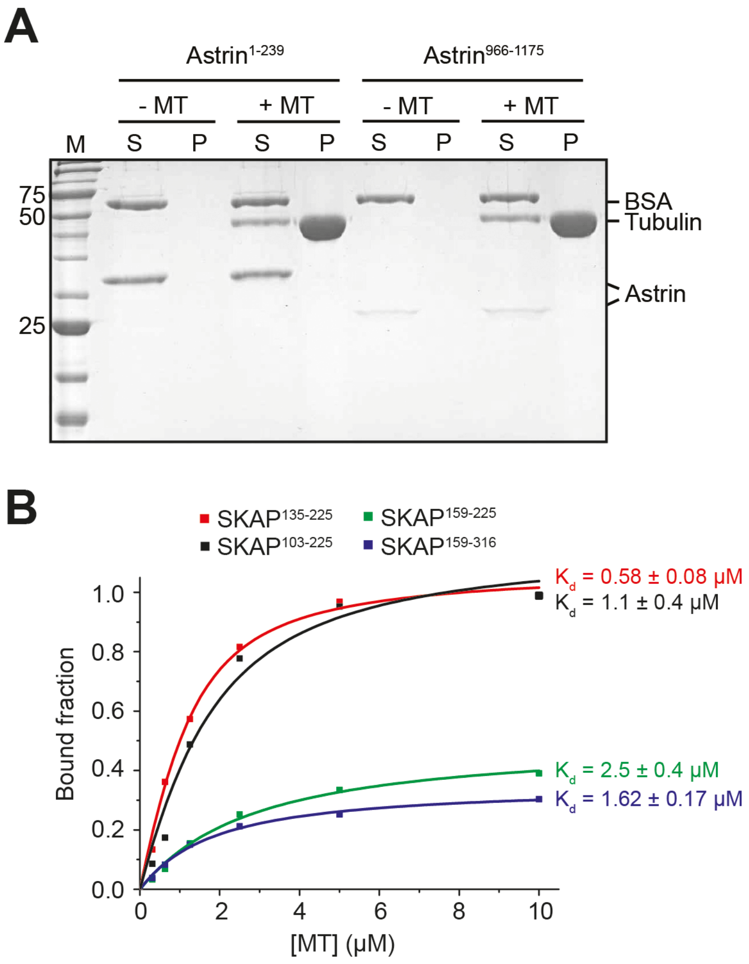

Supplementary Fig. 2

Supplementary Figure 2. Astrin$^{1\text{-}239}$, Astrin$^{966\text{-}1175}$ and SKAP$^{226\text{-}316}$ are dispensable for microtubule binding of the SKAP:Astrin complex. (**A**) Representative SDS-PAGE of microtubule co-sedimentation assay with 3 µM taxol-stabilized microtubules (MT) and 1 µM Astrin$^{1\text{-}239}$ or Astrin$^{966\text{-}1175}$. S: soluble fraction, P: pellet fraction, M: molecular weight marker. (**B**) Quantification and fitting analysis of microtubule co-sedimentation assays with 0-10 µM taxol-stabilized microtubules and 1 µM SKAP$^{135\text{-}225}$, SKAP$^{103\text{-}225}$, SKAP$^{159\text{-}225}$ or SKAP$^{159\text{-}316}$.

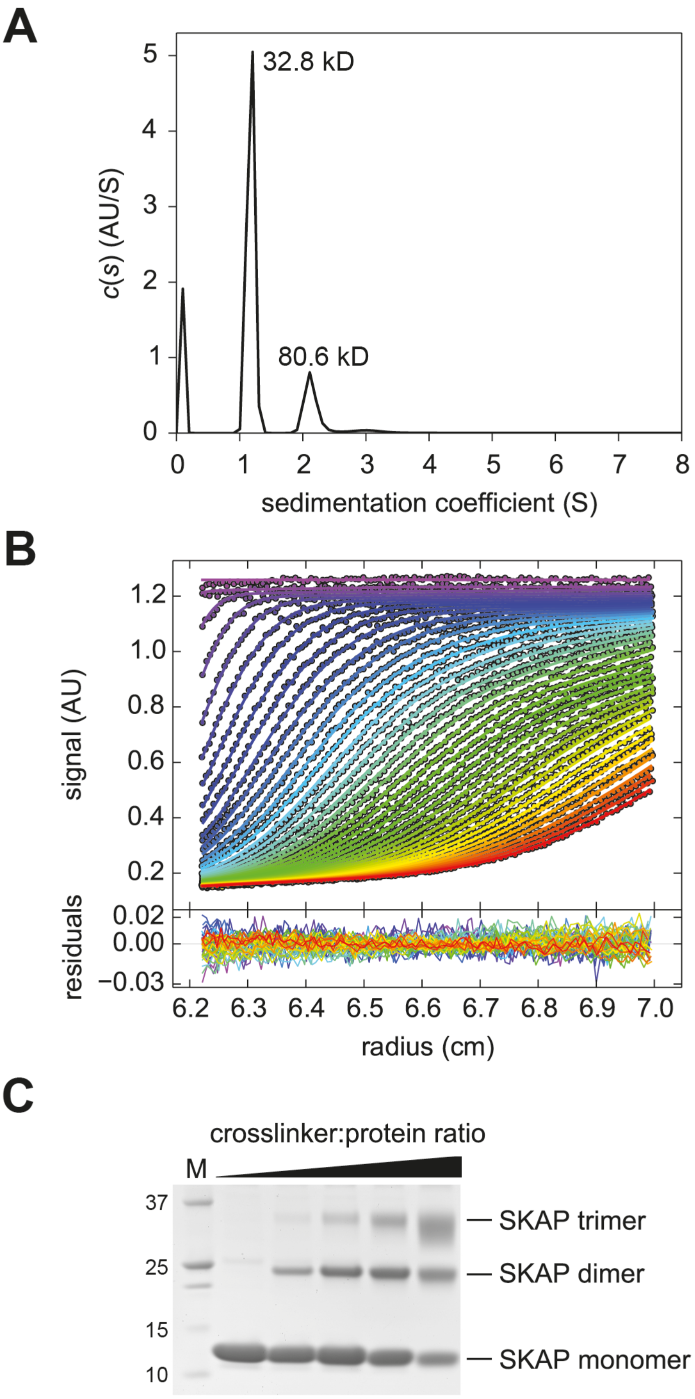

Supplementary Fig. 3

Supplementary Figure 3. SKAP$^{135\text{-}225}$ forms a trimer. (**A**) The best-fit continuous-size c(*S*) distribution of SKAP$^{135\text{-}225}$. (**B**) Top, sedimentation velocity absorbance profiles of SKAP$^{135\text{-}225}$. Bottom, residuals of the fit showing the deviation of the c(*S*) model from the observed signals. (**C**) SDS-PAGE of cross-linking reaction with SKAP$^{135\text{-}225}$ in presence of increasing amounts of crosslinker BS2G.

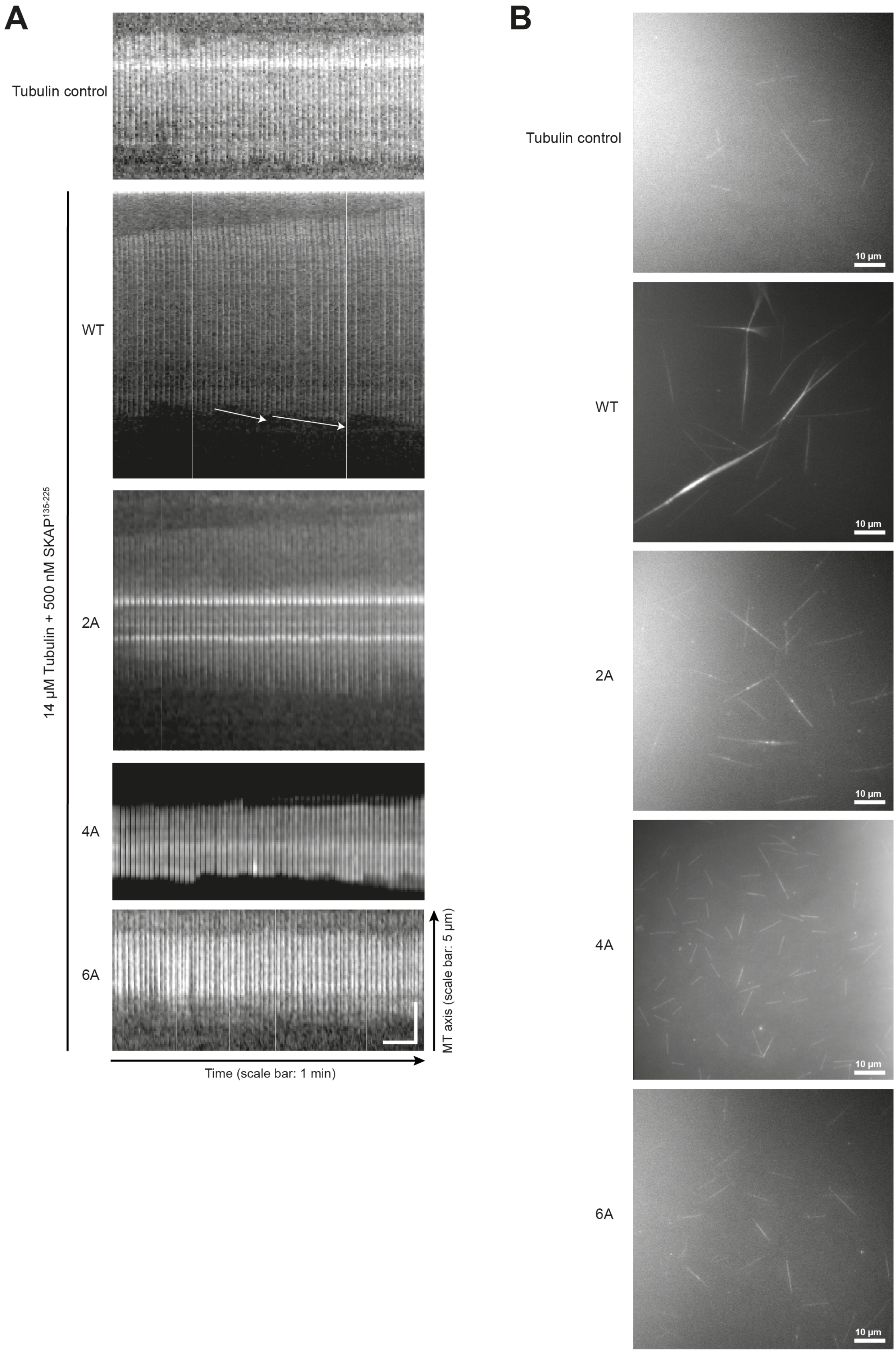

Supplementary Fig. 4

Supplementary Figure 4. SKAP promotes Tubulin polymerization and bundling *in vitro*. (**A**) Kymographs of microtubules observed in TIRF experiments in absence or presence of 500 nM SKAP$^{135\text{-}225}$ wild type or indicated mutants. As an example for growth rate calculation, two analyzed growth events are highlighted for the microtubule depicted for the SKAP$^{135\text{-}225}$ wild type. This shows a growth of 1.43 μm in 1.67 min (left arrow) and 1.37 μm in 2.30 min (right arrow) between sequent catastrophe events, which results in calculated growth rates of 0.86 μm/min and 0.60 μm/min, respectively. (**B**) Representative TIRF images of microtubules in absence or presence of 500 nM SKAP$^{135\text{-}225}$ wild type or indicated mutants. The image for SKAP$^{135\text{-}225}$ wild type shows microtubule bundling by SKAP.

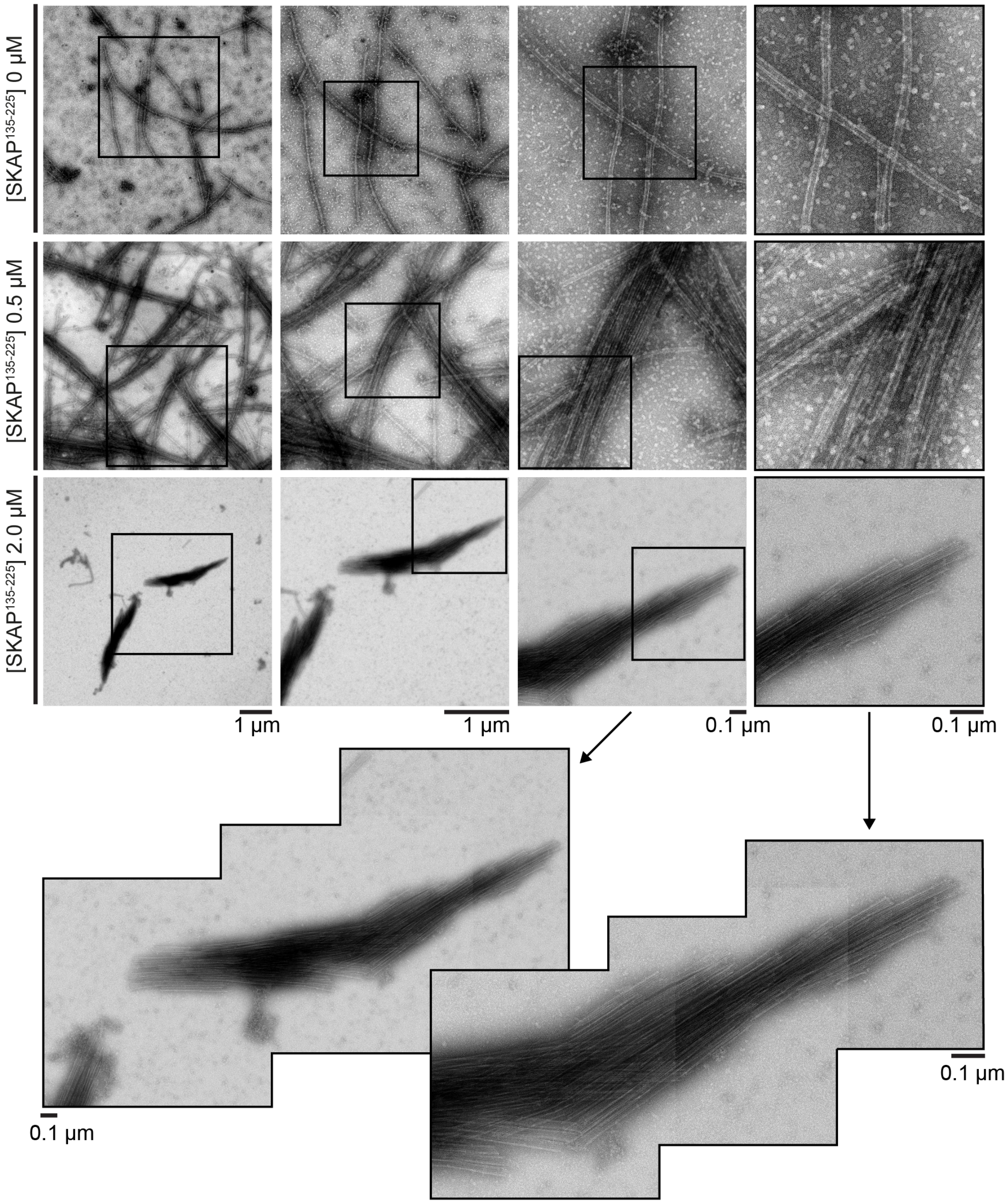

Supplementary Fig. 5

Supplementary Figure 5. SKAP promotes microtubule bundling *in vitro*. Representative electron micrographs of negative-stain Taxol-stabilized microtubules in presence of 0-2 µM SKAP$^{135-225}$. Micrographs in the second and fourth column are shown in Figure 4H.

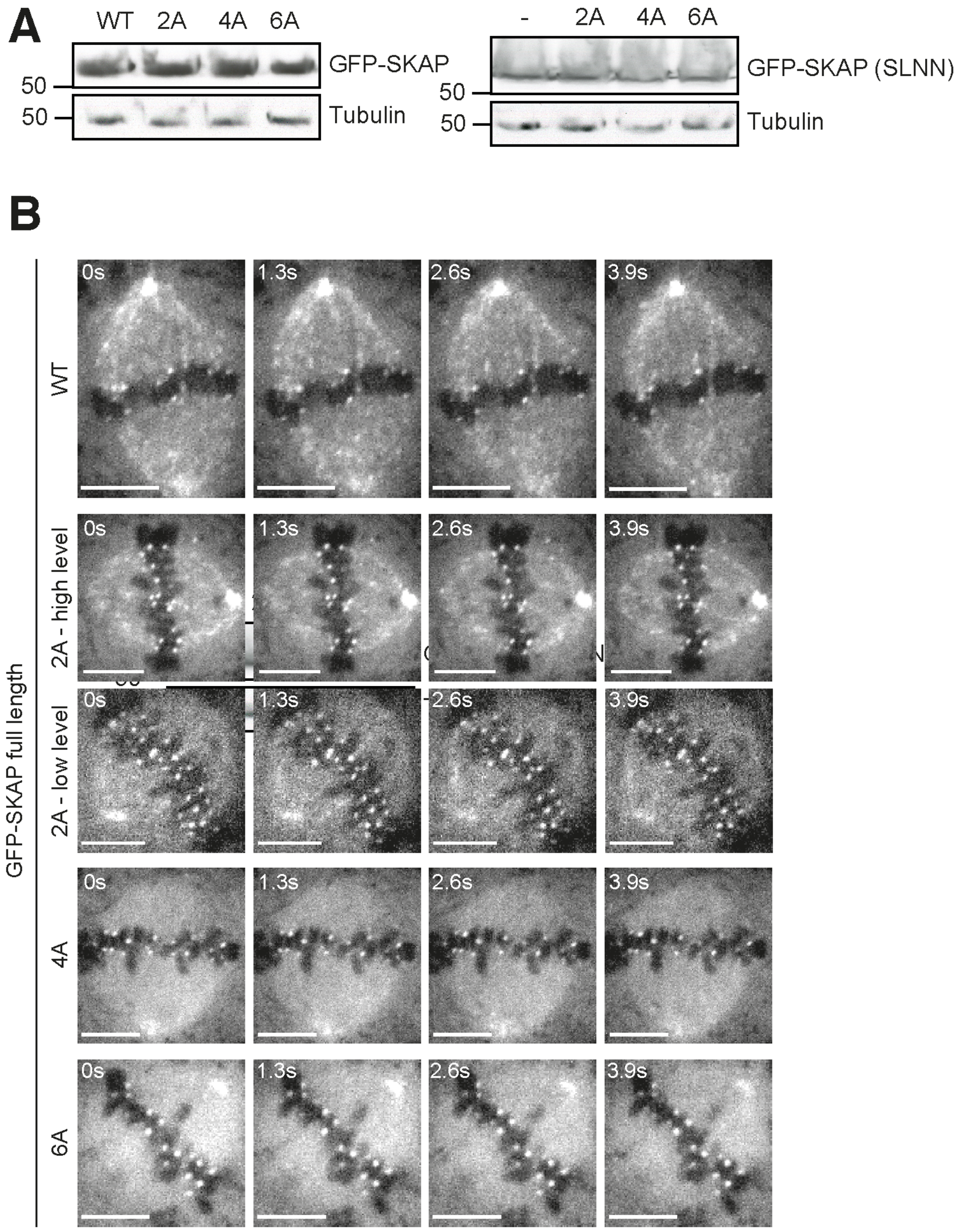

Friese et al. 2015
Figure S6

Supplementary Figure 6. Control of GFP-SKAP expression levels. A) Western blots of lysates of stable Flp-In T-REx cell lines expressing GFP-SKAP full-length wild type (WT) or indicated mutants showing approximately equal expression of wild type and mutant SKAP sequence after induction with doxycycline for 24 h. Blots were stained for GFP and α-Tubulin, respectively. Full blots are shown in Supplementary Figure 9. **B**) Live cell imaging of stable Flp-In T-REx cell lines expressing GFP-SKAP full-length

wild type (WT) or 2A, 4A, and 6A mutants. Experiments with WT and 6A SKAP have already been shown in Figure 5C. Cells were treated with doxycycline for 24 h prior to imaging. Scale bar: 5 µm.

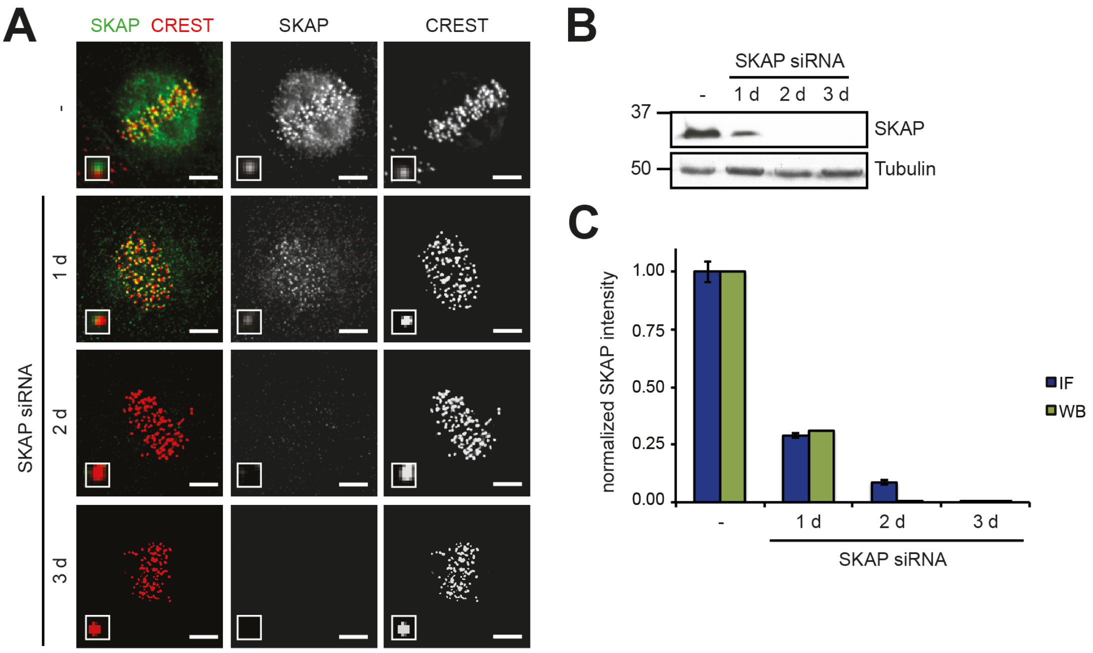

Supplementary Fig. 7

Supplementary Figure 7. Documentation of endogenous SKAP depletion by RNAi. (**A**) Representative immunofluorescence images of Flp-In T-REx cells treated with SKAP siRNA for 1, 2 or 3 d. Scale bar: 5 µm. (**B**) Western blots of lysates of Flp-In T-REx cells treated with SKAP siRNA for 1, 2 or 3 days. Blots were stained for SKAP and α-Tubulin, respectively. Full blots are shown in Supplementary Figure 9. (**C**) Quantification of SKAP intensity in immunofluorescence (IF, n ≥ 100) and western blots (WB, n = 1) shown in (**A**) and (**B**).

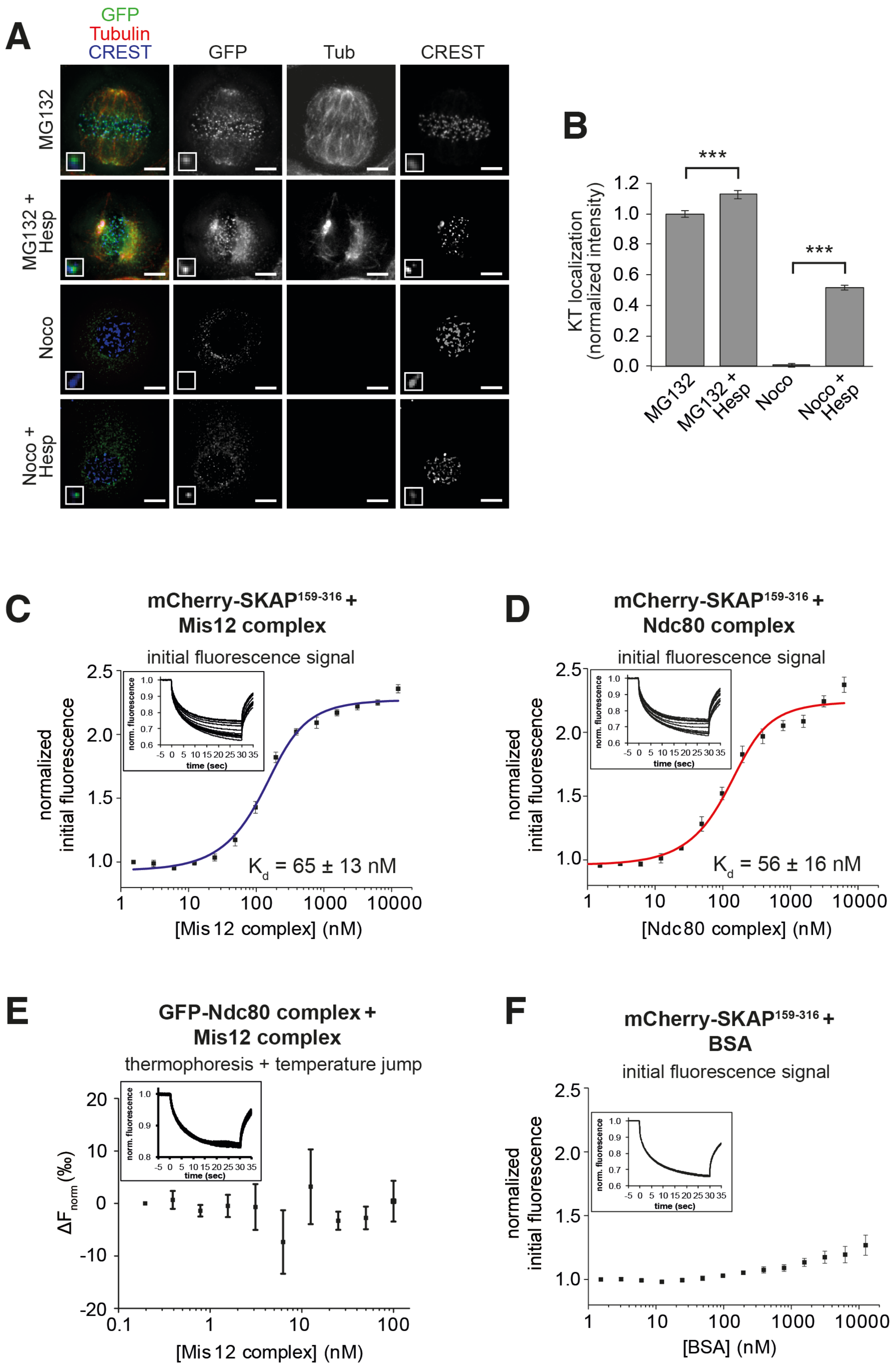

Supplementary Fig. 8

Supplementary Figure 8. SKAP localizes to kinetochores in the absence of microtubules. (**A**) Representative immunofluorescence images of HeLa cells treated with MG132 or nocodazole for 3 h and Hesperadin for 1.5 h prior to fixation. Scale bar: 5 μm. (**B**) Quantification of endogenous SKAP signals at kinetochores for immunofluorescence images shown in (**A**) (mean ± s.e.m., n ≥ 140, *t*-test: *** indicates $p < 0.001$). (**C**) Quantification of microscale thermophoresis (MST) assays with mCherry-SKAP$^{159\text{-}316}$ and the Mis12 complex. The graphs display fluorescence changes normalized to the initial fluorescence (mean ± s.e.m., n = 4). MST-traces are shown in insets. (**D**) Quantification of MST assays with mCherry-SKAP$^{159\text{-}316}$ and the Ndc80 complex. The graphs display fluorescence changes normalized to the initial fluorescence (mean ± s.e.m., n = 4). MST-traces are shown in insets. (**E**) Quantification of MST assays with GFP-tagged Ndc80 complex and the Mis12 complex. The graphs represent thermophoresis and temperature jump signals (mean ± s.e.m., n = 3). (**F**) Control experiment of a titration of mCherry-SKAP$^{159\text{-}316}$ with BSA demonstrates lack of unspecific signals.

Western blots in Fig. S6 A

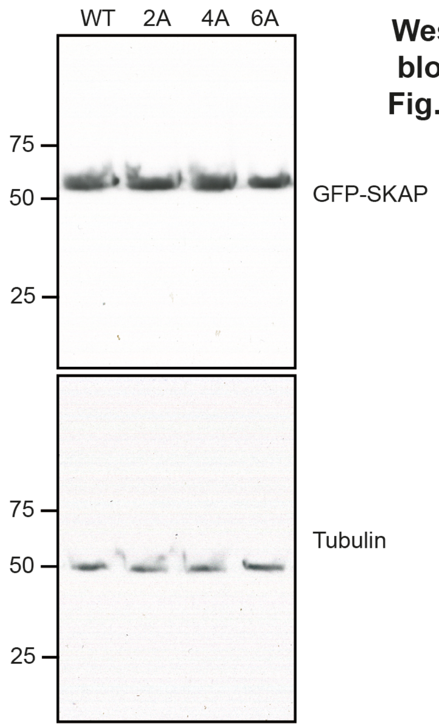

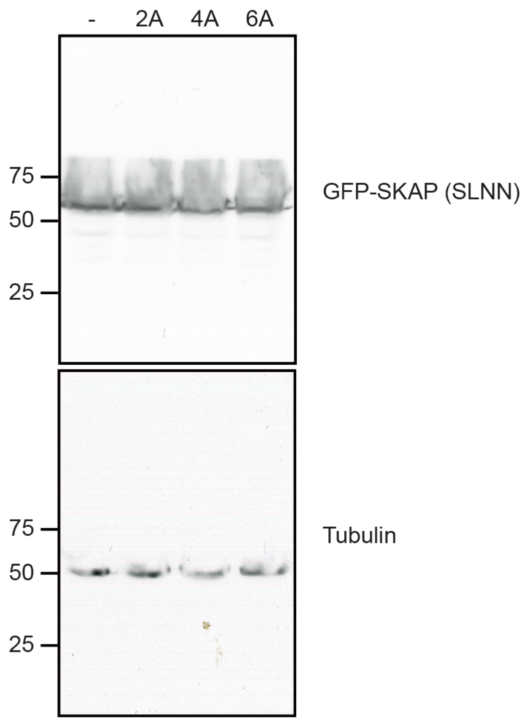

Western blots in Fig. S7 B

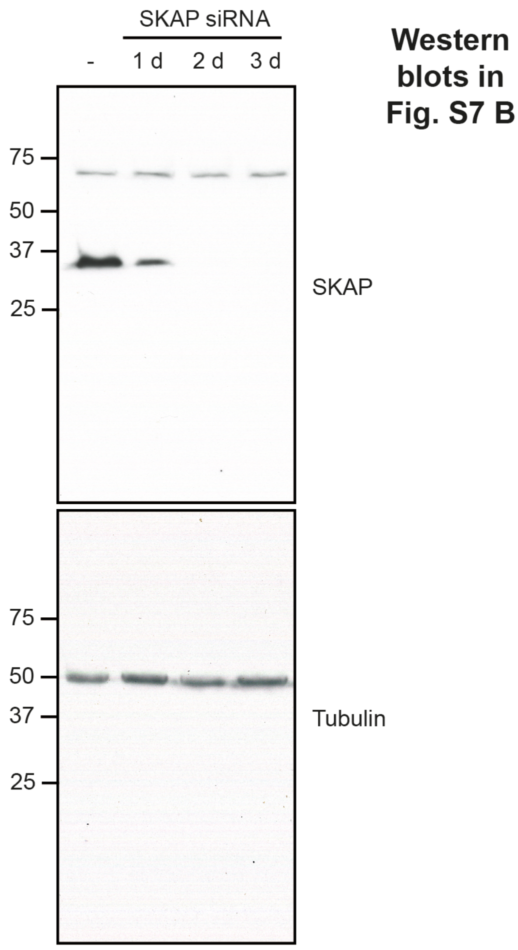

Supplementary Fig. 9

Supplementary Figure 9. Full scans of western blots discussed in this work

Supplementary Table 1

Primer sequences for constructs described in the main text (sequences in bold are complementary to gene sequence)

**construct**	**forward primer**	**reverse primer**	**destination vector**
SKAP$^{135-225}$	AAAAAAGGATCC**GTTACAAAAATCACCAAACTGAGACGAGAG**	AAAAAAGTCGACTTA**GCCCTTGCTCTCCAAAATTGC**	pGEX-6p-2rbs
SKAP$^{1-158}$	AAAAAAGGATCC**ATGGCGGCTCCCGAAG**	AAAAAAGTCGACTTA**ATTCCTTCTGGTGGCAGTG**	pGEX-6p-2rbs
SKAP$^{159-225}$	AAAAAAGGATCC**ATGGTCAGAAAAGGCTACAAACCACTG**	AAAAAAGTCGACTTA**GCCCTTGCTCTCCAAAATTGC**	pGEX-6p-2rbs
SKAP$^{226-316}$	AAAAAAGGATCC**ATGCTTGATCCAGCTTTAGGCAGTG**	AAAAAAGTCGAC**TTACATTTCTAATAGCTGCTCCATTTCCTTAAGGG**	pGEX-6p-2rbs
SKAP$^{159-316}$	AAAAAAGGATCC**ATGGTCAGAAAAGGCTACAAACCACTG**	AAAAAAGTCGAC**TTACATTTCTAATAGCTGCTCCATTTCCTTAAGGG**	pGEX-6p-2rbs
SKAP$^{159-316}$	AAAAAACTCGAG**ATGGTTCGTAAAGGTTATAAACCGCTGTCG**	AAAAAAATGCAT**TTACATTTCCAGCAGCTGTTCC**	pFL
Astrin$^{1-239}$	AAAAAAGGATCC**ATGTGGCGTGTTAAAAAACTGAG**	AAAAAAGTCGACTTA**TGCATTGCTTTCGCTCGG**	pGEX-6p-2rbs
Astrin$^{1-481}$	AAAAAAGGATCC**ATGTGGCGTGTTAAAAAACTGAG**	AAAAAAGTCGACTTA**GGTAATACCGCTATGACTGGTATC**	pGEX-6p-2rbs
Astrin$^{966-1175}$	AAAAAAGGATCC**ATGGAAGAGTCACTGGCC**	AAAAAAGTCGACTTA**AATACTCAGCAGGGTTTTATAGATGTGC**	pGEX-6p-2rbs
Astrin$^{482-850}$-His$_6$	AAAAAAGTCGAC**ATGAATAAACTGCAGCACCTGAAAGAAAG**	AAAAAAAAGCTTTTAGTGATGGTGATGGTGATG**GGCGGTCAGATTTTCCAC**	pFL
